# Adaptive Evolution of the Lactose Utilization Network in Experimentally Evolved Populations of *Escherichia coli*

**DOI:** 10.1371/journal.pgen.1002444

**Published:** 2012-01-12

**Authors:** Selwyn Quan, J. Christian J. Ray, Zakari Kwota, Trang Duong, Gábor Balázsi, Tim F. Cooper, Russell D. Monds

**Affiliations:** 1 Bio-X Program, Department of Chemical and Systems Biology, Stanford University School of Medicine, Stanford, California, United States of America; 2 Department of Systems Biology–Unit 950, The University of Texas MD Anderson Cancer Center, Houston, Texas, United States of America; 3 Department of Biology and Biochemistry, University of Houston, Houston, Texas, United States of America; University of Toronto, Canada

## Abstract

Adaptation to novel environments is often associated with changes in gene regulation. Nevertheless, few studies have been able both to identify the genetic basis of changes in regulation and to demonstrate why these changes are beneficial. To this end, we have focused on understanding both how and why the lactose utilization network has evolved in replicate populations of *Escherichia coli*. We found that *lac* operon regulation became strikingly variable, including changes in the mode of environmental response (bimodal, graded, and constitutive), sensitivity to inducer concentration, and maximum expression level. In addition, some classes of regulatory change were enriched in specific selective environments. Sequencing of evolved clones, combined with reconstruction of individual mutations in the ancestral background, identified mutations within the *lac* operon that recapitulate many of the evolved regulatory changes. These mutations conferred fitness benefits in environments containing lactose, indicating that the regulatory changes are adaptive. The same mutations conferred different fitness effects when present in an evolved clone, indicating that interactions between the *lac* operon and other evolved mutations also contribute to fitness. Similarly, changes in *lac* regulation not explained by *lac* operon mutations also point to important interactions with other evolved mutations. Together these results underline how dynamic regulatory interactions can be, in this case evolving through mutations both within and external to the canonical lactose utilization network.

## Author Summary

Differences in gene regulation underlie many important biological processes and are thought to be important for the adaption of organisms to novel environments. Here we focus on the regulation of a group of well-studied genes, the *lac* operon, that control the utilization of lactose sugar, and we examine how their regulation changes during the adaptation of populations of *Escherichia coli* bacteria to environments that differ only in the presence of lactose. We find that *lac* operon regulation is altered in almost all populations that evolve in the presence of lactose and identify two classes of mutations that explain a large part of this change and that confer significant fitness benefits. Interestingly, our study indicates that other mutations, lying outside of the commonly recognized control region, cause new regulation of the *lac* operon. Together these findings reinforce the importance of changes in gene regulation during evolution and suggest that the biological basis of these changes can be complex and involve novel interactions between genes.

## Introduction

Changes in gene regulation are an important and common cause of adaptation. Support for this comes from bioinformatic evidence that changes in regulatory elements are associated with presumably adaptive phenotypic changes (reviewed in [1], [2]), comparative experimental studies [3] and from experimental evolution studies, which often find regulatory changes occurring during adaptation to novel environments [4]–[12]. Indeed, in some of these cases direct links have been established between regulatory changes and adaptation [6]–[8], [10]. These experiments directly demonstrate that small and local regulatory changes can significantly contribute to adaptation. In most cases, however, the physiological basis for selection of regulatory changes is unknown.

Previously, we described the evolution of populations of *Escherichia coli* in defined environments that differed only in the number and presentation of the limiting resource [13]. Populations were evolved in environments supplemented with a single limiting resource or combinations of two limiting resources either presented together or fluctuating daily. These populations adapted to the environments in which they were evolved and this adaptation was, at least to some extent, environment-specific [13]. Here, we focus on a subset of 24 populations that evolved in environments supplemented with glucose and/or lactose and examine changes in the regulation of the *lac* operon in these populations.

Several attributes make the *lac* operon a good candidate in which to observe regulatory changes and relate them to their physiological and fitness effects. First, the costs and benefits of *lac* operon expression are environmentally dependent. Expression of the *lac* operon is necessary for utilization of lactose for growth, but expression in the absence of lactose can impose a significant cost [14]–[16]. Second, the molecular components of the lactose utilization network are well characterized and their activity can be measured *in vivo*. The ability to assay changes in the *lac* regulatory network ‘output’ provides a means to identify and test activity of evolved regulatory changes. Third, the *lac* operon has been the subject of much theoretical work, leading to the development of mathematical models to explain important features of *lac* operon physiology [14], [17]–[22] and evolution [16], [23], [24]. Fourth, the utility of the *lac* operon for examining evolution of regulatory changes has been demonstrated experimentally. For example, *lac* operon constitutive [25], [26], loss of function [14] and duplication [11] mutants have been recovered following growth in different selective environments, demonstrating that *lac* operon regulation is evolutionarily flexible and can be a target of selection. It has even been possible to predict the evolution of regulatory changes on the basis of their expected fitness effects. Dekel and Alon (2005) used a cost-benefit analysis to predict the optimum expression level of the *lac* operon in different inducer concentration environments. They found that populations selected in environments containing a high level of gratuitous inducer, but various concentrations of lactose, generally evolved to regulate expression of the *lac* operon to the predicted level.

In this study, we examine changes in *lac* operon regulation associated with selection in environments differing in the presentation of its natural substrate and inducer, lactose, and repressor, glucose. In addition to quantitatively characterizing the changes that have occurred, we examine the genetic and demographic basis for selection of different modes of *lac* regulation. We find that regulatory changes in the *lac* operon evolved in many replicate populations selected in environments containing lactose. Much, but not all, of these changes were due to mutations in the LacI repressor or its major operator binding site within the *lac* promoter. By themselves, these mutations conferred significant fitness benefits in all of the evolution environments that contained lactose. We also present evidence for interactions between *lac* mutations and mutations in genes outside of the canonical *lac* utilization network and show that these interactions impact *lac* operon regulation and fitness. Finally, operator and repressor mutations fixed at different frequencies in different selective environments, although the selective basis of this is currently not known.

## Results

### Qualitative characterization of evolved changes to *lac* operon regulation

**Figure 1:**
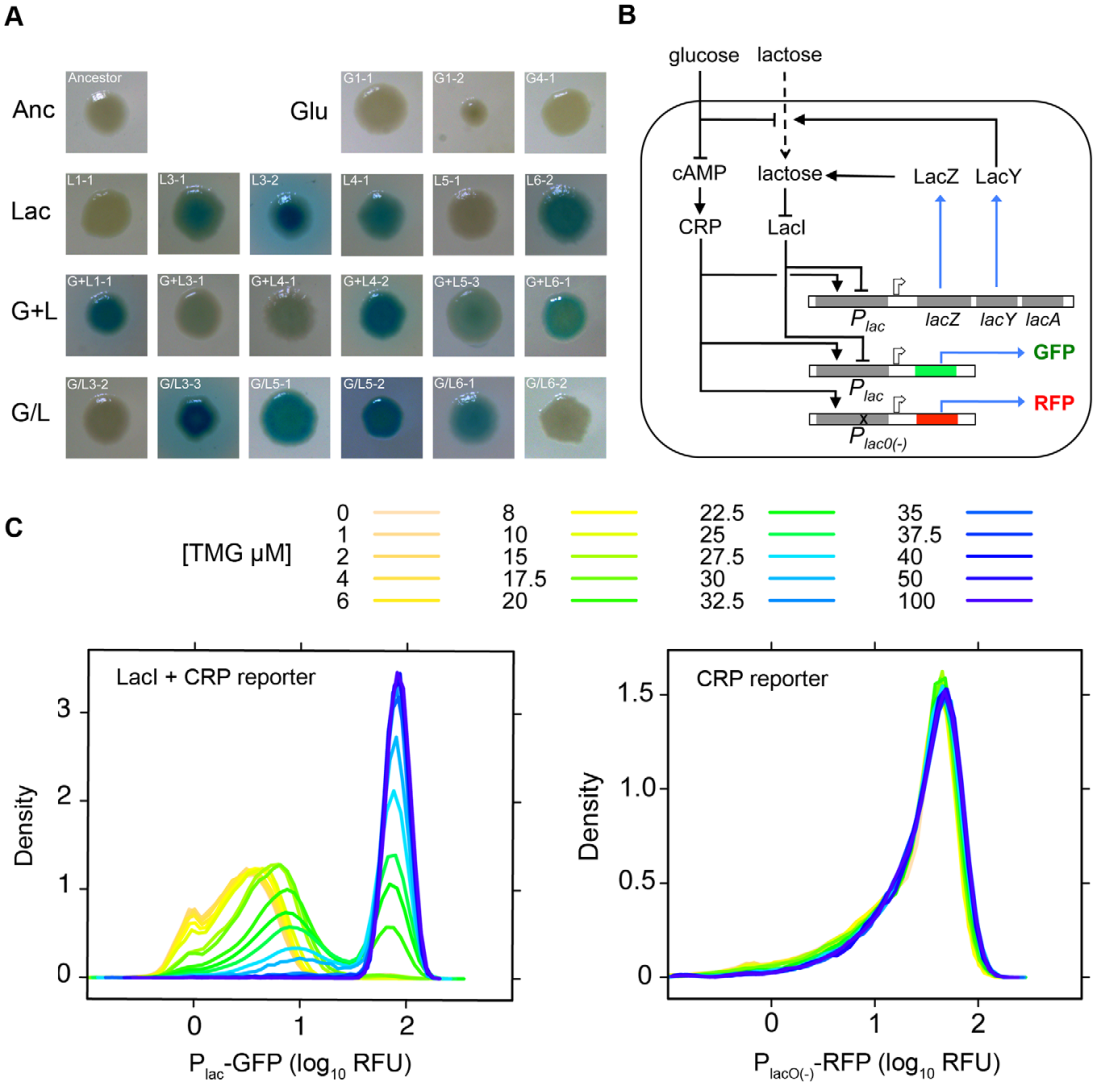
Characterization of *lac* operon regulation. A) Examples of the range of evolved LacZ activity phenotypes present in the four evolution environments. Degree of blue coloration on TGX plates gives a qualitative measure of LacZ activity. B) Schematic of *lac* operon and reporters used to measure *lac* operon regulation. *lacZ* encodes the β-galactosidase responsible for lactose catabolism and *lacY* encodes a lactose permease. The expression of *lacZYA* is directly controlled by LacI and CRP. LacI is a negative regulator, binding to operator sites within the *lacZYA* promoter (P_lac_). LacI binding is inhibited by lactose and gratuitous inducers, such as TMG. CRP is a positive regulator, activating *lacZYA* expression when cAMP levels are elevated in response to low glucose concentrations. High levels of glucose also repress *lac* expression by inhibiting import of lactose through LacY. Two reporters were designed to measure LacI and CRP inputs into *lac* operon regulation. The native *lac* promoter drives expression of GFP and is subject to regulation by both LacI and CRP. A second reporter utilizes a mutant *lac* promoter that cannot bind LacI to drive expression of DsRedExpress2. This reporter is only subject to regulation by CRP. Solid lines indicate positive (arrows) and negative (blunt arrow) regulatory interactions; dotted lines indicate the transfer of metabolites; blue lines indicate the production of proteins; open arrows indicate expression start sites. Figure adapted from Ozbudak et al. 2004. C) Ancestral inducer response profile. Shown are flow cytometry histograms for the ancestor grown in a range of TMG concentrations. P_lac_-GFP and P_lac(O-)_-RFP measurements were taken simultaneously from the same cultures.

Previously we reported propagation of 24 replicate populations of *E. coli* B REL606 for 2000 generations in one of four environments that differed only in the concentration and/or presentation of glucose and lactose [13]. Environments used were glucose (Glu), lactose (Lac), glucose and lactose presented simultaneously (G+L) or alternating daily between glucose and lactose (G/L). To determine whether regulation of the *lac* operon had changed during the evolution of our experimental populations, we screened ≥1000 clones from each population on LacZ indicator plates (see Materials and Methods). All six Glu populations had LacZ phenotypes that were indistinguishable from the ancestor. By contrast, in all other evolution environments at least some replicate populations showed clear changes in LacZ activity (Lac, 3 of 6; G+L, 6 of 6; G/L, 6 of 6) (Table 1, Figure 1A). Some of these populations also had within-population variation in LacZ activity and colony morphology. To facilitate molecular and physiological studies of these changes we identified and isolated 46 clones (at least one from each evolved population) that encompassed the range of LacZ activity and morphology types present across all populations (Table 1). These clones were used in all subsequent analyses.

**Table 1:** Characterization of evolved clones.

	Clone name	Indicator plate phenotype	Frequency	Genotype	Regulatory class
**Glu**	G1-1	large, white, smooth margin	0.668	wt	bimodal
	G1-2	small white	0.332	wt	bimodal
	G2-1	white	1.000	wt	bimodal
	G3-1	large white	1.000	wt	bimodal
	G4-1	white	1.000	wt	bimodal
	G5-1	white	1.000	wt	bimodal
	G6-1	white	1.000	wt	bimodal
**Lac**	L1-1	cream, smooth margin	1.000	wt	bimodal
	L2-1	cream, smooth margin	1.000	wt	bimodal
	L3-1	large, pale blue	0.290	*lacI*-(ΔTGGC)	constitutive
	L3-2	dark blue, smooth margin	0.697	*lacI*-(ΔTGGC)	constitutive
	L3-3	small, dark blue, smooth margin	0.012	*lacI*-(ΔTGGC)	constitutive
	L3-4	very small, white, translucent	0.001	*lacI*-(ΔTGGC)	constitutive
	L4-1	large, pale blue	0.999	*lacI*-(ΩTGGC)	constitutive
	L4-2	white	0.001	*lacI*-(ΩTGGC)	constitutive
	L5-1	large, cream	1.000	wt	bimodal
	L6-1	small, bright blue, diffuse margin	0.405	*lacI*-(ΩTGGC)	constitutive
	L6-2	blue, diffuse margin	0.595	*lacI*-(ΩTGGC)	constitutive
**G+L**	G+L1-1	small, uniform dark blue	0.494	*lacI*-(ΩTGGC)	constitutive
	G+L1-2	large, flat, light blue	0.506	*lacI*-(ΩTGGC)	constitutive
	G+L2-1	large, faint blue	1.000	*lacO1*-G9T	lower threshold
	G+L3-1	large, faint blue, diffuse margin	1.000	*lacO1*-G11A	lower threshold
	G+L4-1	large, faint blue	0.066	*lacO1*-G5A	lower threshold
	G+L4-2	large, blue	0.934	*lacI*-(ΩTGGC)	constitutive
	G+L5-1	large, flat, faint blue	0.127	*lacO1*-G11A	lower threshold
	G+L5-2	small, blue, smooth margin	0.015	*lacO1*-G11A	lower threshold
	G+L5-3	large, flat, pale blue	0.858	*lacO1*-G11A	lower threshold
	G+L6-1	blue, smooth margin	0.707	*lacO1*-G5A	lower threshold
	G+L6-2	blue, diffuse margin	0.275	*lacO1*-G5A	lower threshold
**G/L**	G/L1-1	dark blue, smooth margin	0.005	*lacI*-(ΔTGGC)	constitutive
	G/L1-2	blue, smooth margin	0.974	*lacI*-(ΔTGGC)	constitutive
	G/L1-3	light blue, irregular margin	0.021	*lacI*-(ΔTGGC)	constitutive
	G/L2-1	dark blue, smooth margin	0.052	*lacI*-(ΔTGGC)	constitutive
	G/L2-2	flat, blue, smooth margin	0.946	*lacI*-(ΔTGGC)	constitutive
	G/L2-3	large, flat, white	0.002	wt	bimodal
	G/L3-1	blue, smooth margin	0.990	*lacI*-(ΩTGGC)	constitutive
	G/L3-2	flat, white, smooth margin,	0.009	wt	bimodal
	G/L3-3	small, dark blue, irregular shape	0.001	*lacI*-(ΩTGGC)	constitutive
	G/L4-1	small, dark blue, smooth margin	0.300	*lacI*-(ΩTGGC)	constitutive
	G/L4-2	pale blue, large, irregular shape	0.638	*lacI*-(ΩTGGC)	constitutive
	G/L4-3	no blue, irregular margin & shape	0.061	*lacI*-(L71Q)	constitutive
	G/L5-1	large, blue, smooth margin	0.461	No Seq	constitutive
	G/L5-2	small blue, round margin	0.536	No Seq	constitutive
	G/L5-3	large, pale blue	0.003	No Seq	constitutive
	G/L6-1	large, pale blue, diffuse margin	0.996	*lacI*-(ΔTGGC)	constitutive
	G/L6-2	large, white, irregular shape	0.004	wt	bimodal

### Quantitative analysis of evolved changes to *lac* operon regulation

**Figure 2:**
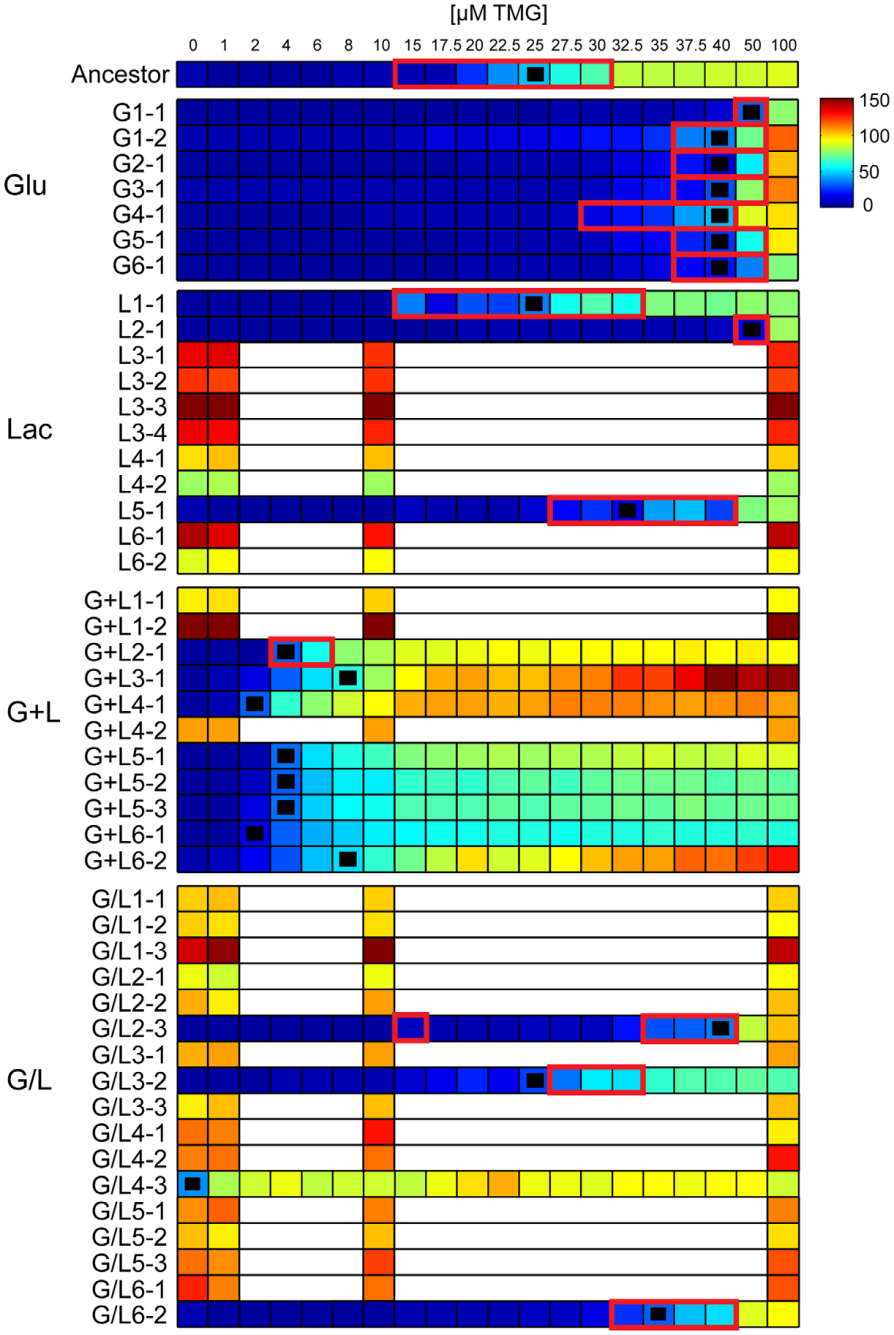
Inducer response profiles of evolved clones. Heat maps show the mean response of the P_lac_-GFP reporter (RFU) for evolved clones at different concentrations of inducer (TMG). Black squares indicate the concentration of TMG that gave half maximal expression (TMG_½Max_) and red outlines indicate the presence of bimodal expression within the population. Inducer responses for clones with constitutive *lac* operons were only generated with four TMG concentrations because *lac* expression was independent of inducer concentration such that finer resolution measurements were redundant.

To characterize evolved changes in *lac* operon regulation, we used a dual fluorescence reporter system to independently quantify with single cell resolution the activity of the major transcriptional regulators of the *lac* operon, LacI and CRP. LacI and CRP bind the *lac* promoter at different locations, repressing and activating transcription of the *lac* operon, respectively (Figure 1B). The ancestor and each of the 46 evolved clones were transformed with P_lac_-GFP (LacI and CRP reporter) and P_lacO(-)_-RFP (CRP reporter) constructs. For each strain we used flow cytometry to measure the population distribution of steady-state GFP and RFP expression over a range of thiomethyl-galactoside (TMG) concentrations (TMG is a non-metabolizable inducer of the *lac* operon). The P_lac_-GFP reporter captured several key features of *lac* regulation. The inducer response of the ancestor is ultra-sensitive, showing a sharp transition from low to high expression states as a function of TMG concentration and is bimodal, with populations showing a mix of non-induced and fully induced cells at intermediate levels of TMG [19], [27], [28] (Figure 1C, Figure 2). The CRP-only reporter (P_lacO(-)_-RFP) shows a unimodal distribution with a constant mean over the range of TMG concentrations, confirming that CRP activity of the ancestor is independent of LacI activity and *lac* operon expression state (Figure 1C). To facilitate comparisons between the ancestral and evolved genotypes, we quantified three aspects of *lac* regulation: the TMG concentration required for half maximal population expression (TMG_½ Max_), the range of TMG concentrations causing a bimodal population response and the fully induced (maximum) steady state level of *lac* expression. For the ancestor, TMG_½ Max_ is 25 µM, the range of bimodality is between 15–30 µM TMG, and the level of P_lac_-GFP at full induction (100 µM TMG) is ∼84 RFU (Figure 2).

**Figure 3:**
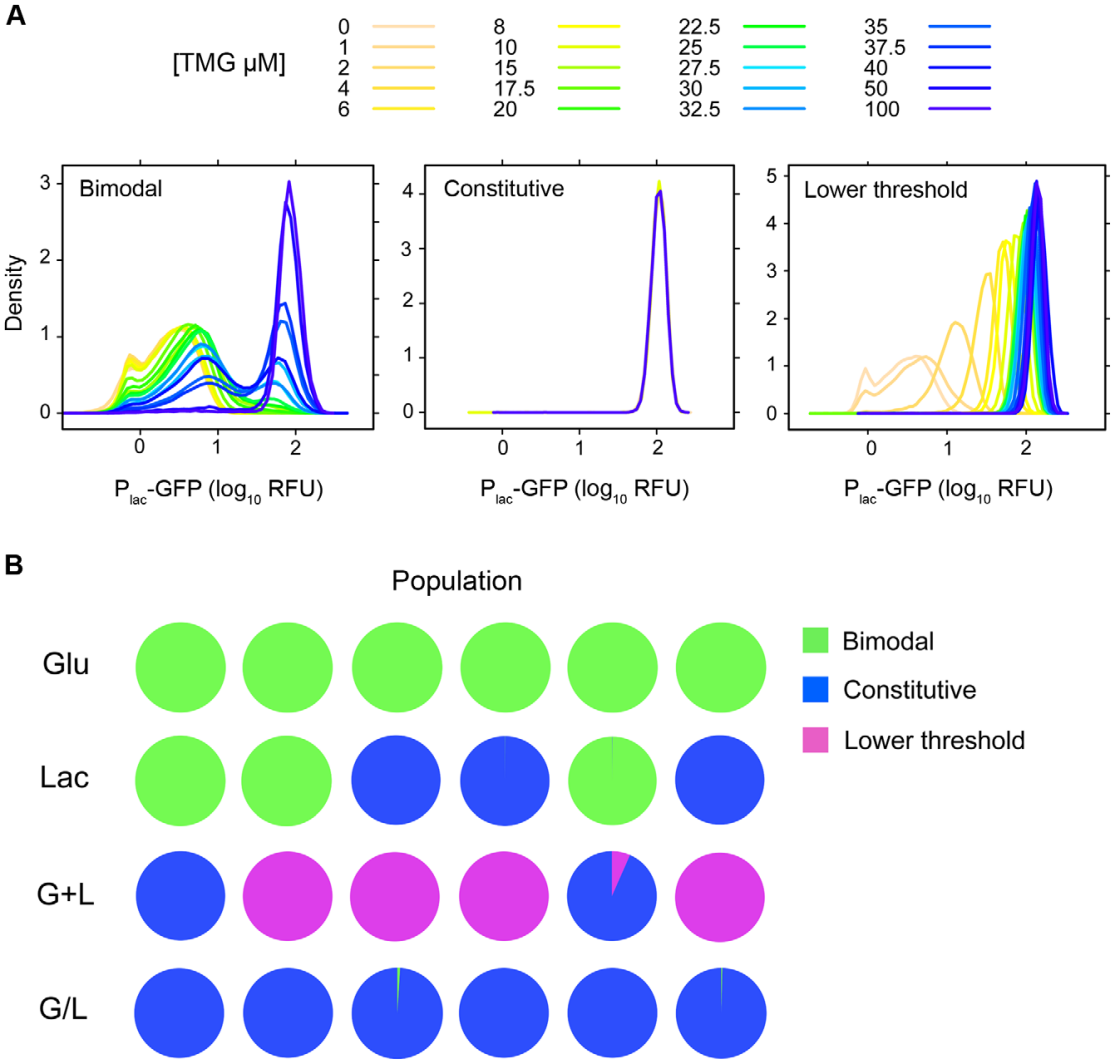
Inducer response classes and association with evolution environment. A) Inducer response classes for evolved clones. 1) Bimodal with a higher threshold for *lac* induction, 2) Constitutive with expression levels that are independent of inducer, 3) Lower threshold for *lac* induction with graded response to inducer. Inducer response histograms are shown for clones representative of each class (Bimodal, L5-1; Constitutive, G/L5-1; Lower threshold, G+L3-1). B) Distribution of inducer response types by population and evolution environment. Pie charts show the fraction of clones with each inducer response type (Bimodal, Constitutive, Lower threshold) within a given population.

Evolved changes in the inducer response profiles were common and fell into three broad classes (Figure 2). 1) Constitutive: operationally defined as mean P_lac_-GFP expression varying by less than 2-fold across the range of tested TMG concentrations. Constitutive clones were observed in at least some populations of each of the treatments containing lactose (Lac, G+L and G/L), but were not observed in any of the Glu evolved populations. 2) Lower threshold/graded response: increased sensitivity to inducer with TMG_½ Max_ values ranging from 2 µM to 8 µM. In addition, all but one clone with a lower induction threshold (G+L2-1) also evolved a graded response, with a unimodal distribution of P_lac_-GFP expression that increased continuously as a function of TMG concentration. 3) Bimodal: bimodal induction response differing from the ancestor by generally being less sensitive to the inducer, with TMG_½ Max_ values ranging from 25 µM to 50 µM TMG (Figure 2). Higher inducer thresholds were observed for all clones evolved in Glu as well as some clones evolved in Lac and G/L environments. Representative inducer response curves of each class are shown in Figure 3A and the distribution of response types across environments is shown in Figure 3B. Interestingly, clones with a lower threshold response were found exclusively in populations evolved in the G+L environment, fixing in four of the six populations. This pattern is unlikely to occur by chance (Fisher's exact test omitting the polymorphic G+L population, *P* = 0.002). By contrast, populations evolved in the G/L environment were almost exclusively composed of clones with constitutive *lac* expression and populations evolved in Lac had either bimodal or constitutive regulation. All clones evolved in Glu showed a bimodal response type (Figure 2, Figure 3B).

**Figure 4:**
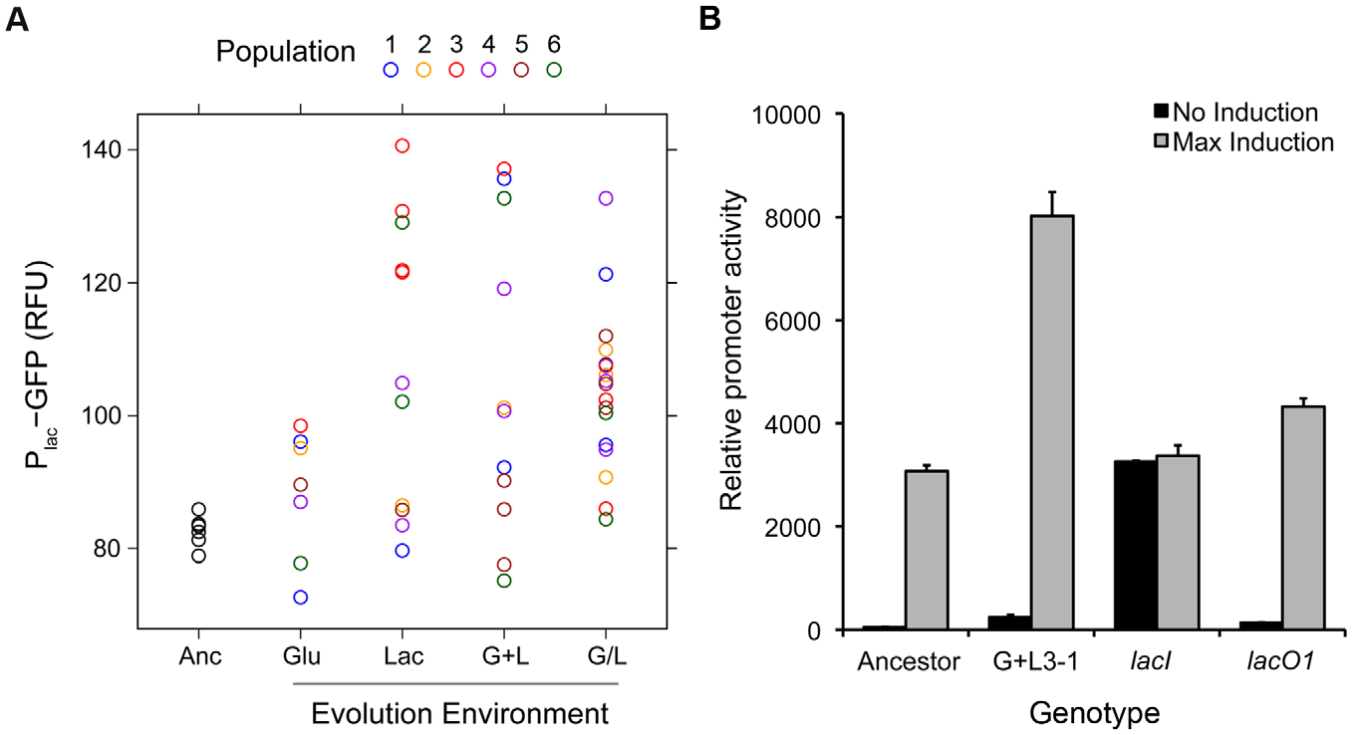
Changes in maximal *lac* expression for evolved clones. A) Steady state P_lac_-GFP levels (RFU) are shown for evolved clones grown in the presence of 100 µM TMG. Clones are divided into categories by evolution environment: Glu, Lac, G+L and G/L. Markers of the same color denote clones recovered from the same evolved population. Data points are the average of two independent replicates. As a reference, data for six independent ancestral (Anc) samples is also shown. B) Native *lac* promoter activity for the ancestor, evolved clone G+L3-1 and the ancestor with *lacI* and *lacO1* mutations. Basal promoter activity was measured for strains grown in the absence of inducer, and maximum promoter activity was measured in the presence of saturating levels of inducer.

The level of P_lac_-GFP expression at maximum induction (100 µM TMG) was higher than the ancestor in 36 of the 46 evolved clones (Figure 2). To examine this observation in more detail, we repeated our measurements of P_lac_-GFP expression in each evolved clone, but this time only in the presence of 100 µM TMG, which enabled us to include all clones in duplicate in the same experimental block (Figure 4A). We again saw a strong trend toward an increase in *lac* expression with 40 of 46 clones having a mean reporter expression level greater than the ancestor. Furthermore, the mean change in expression level of clones evolved in lactose containing environments was significantly higher than the ancestor (two tailed *t*-test with unequal variance: Lac, *P* = 0.008; G+L, *P* = 0.004; G/L, *P*<0.001) but not significantly different for clones evolved in the Glu environment (*P* = 0.242). To verify that changes in the level of our GFP reporter accurately reflected changes in the expression level of the native *lac* operon, we used a direct assay to measure the *lac* promoter activity of a focal evolved clone G+L3-1, which shows an approximate 2-fold increase in maximal P_lac_-GFP expression [29]. We found that expression from the *lac* promoter was significantly higher in the G+L3-1 clone than the ancestor and this increase agreed with our estimate based on flow cytometry (Figure 4B). Measurements taken at two time-points during exponential growth gave similar promoter activity estimates for all strains tested, indicating that promoter activities are representative of the *lac* system at steady state.

Lastly, we measured the expression level of the CRP activity reporter (P_lacO(-)_-RFP) as a function of inducer concentration. All evolved clones showed a unimodal distribution with a mean response that was independent of inducer concentration, suggesting that, similar to the ancestor, evolved clones maintained predominantly non-cooperative interactions between the LacI and CRP regulators and that CRP activity is independent of *lac* expression level (Figure S1).

### Genetic basis of evolved changes in *lac* regulation

**Figure 5:**
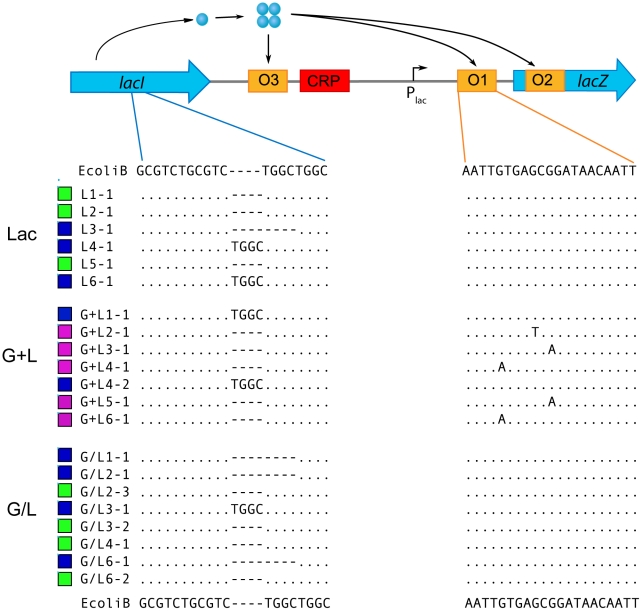
Identification *lac* mutations in evolved clones. Sequence alignments for evolved clones relative to the ancestor are shown for the *lacI* mutation hotspot region (left) and the primary *lacO1* operator (*right*). Clones were chosen that represent the diversity in *lac* genotypes within each population. Colored boxes by sequences indicate the inducer response type for each clone. Bimodal is shown as green, constitutive is shown as blue and lower threshold response is shown as purple. No sequence was obtained for the G/L5 clones. Sequence alignments are not shown for Glu clones since all have wt *lac* regulatory sequences.

To determine the genetic basis of changes to *lac* regulation we first sequenced the main *lac* regulatory regions, *lacI* and P_lac_, of each evolved clone (excluding G/L5 clones whose *lac* regulatory region could not be amplified by PCR). We found that all but one clone classified as constitutive had either a deletion or insertion of a 4 bp sequence within the *lacI* gene, which results in a frame shift (Figure 5). This region of *lacI* has three 4 bp direct repeats and is known to be a mutational hotspot, accounting for ∼75% of all spontaneous *lacI* null mutations [30], [31]. Constitutive clone G/L4-3 had a nonsynonymous mutation in *lacI* conferring a leucine to glutamine change at residue 71. This mutation is predicted to cause a severe defect in the ability of LacI to repress *lac* expression [32]. In addition, we found that all clones that evolved a lower induction threshold contained a single base pair substitution in the primary LacI repressor binding site of the *lac* promoter (*lacO1*). We identified three unique *lacO1* mutations, two of which occurred twice in independent G+L populations (Figure 5). Previous work has demonstrated that all three *lacO1* mutations can reduce the binding efficiency of LacI to the operator, thereby reducing repression of the *lac* operon in the absence of a specific inducer [33]–[36]. Lastly, populations in the bimodal inducer response class did not have any mutations within *lacI* or the *lac* promoter region, even though other aspects of their *lac* regulation, such as the region of bimodalilty and TMG_½Max_ values, were altered (Figure 2).

### Regulation of evolved promoter reporters

The inducer response profiles presented above used a GFP reporter controlled by the ancestral P_lac_ promoter. It is possible that this promoter does not accurately reflect the activity of the mutant promoters that evolved in the G+L isolated strains. To investigate the effect of this difference we constructed a version of the P_lac_-GFP reporter with the *lacO1* ^G11A^ mutation found in the G+L3 and G+L5 populations (P_lacO1_-GFP) and used it to generate inducer response profiles in the G+L3-1 clone (Figure S2A). Inducer response profiles for G+L3-1 with P_lacO1_-GFP are qualitatively similar to those obtained with the P_lac_-GFP reporter, showing a graded inducer response and a lower induction threshold. These characteristics were confirmed with β-Gal enzymatic assays that directly examined mean LacZ activity (Figure S2B).

### Reconstruction of *lac* mutations and inducer response profiles

**Figure 6:**
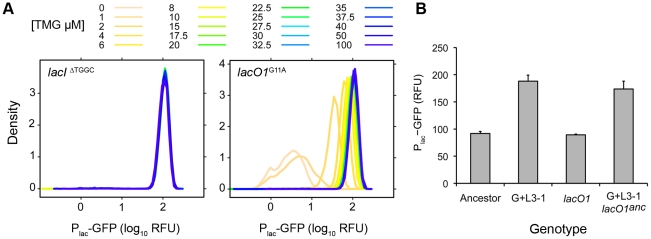
Contribution of *lac* mutations to evolved inducer responses. A) Inducer response histograms for reconstructed *lacI* ^ΔTGGC^ and *lacO1* ^G11A^ mutants. The *lacI* mutation confers constitutive *lac* expression, whereas the *lacO1* mutation confers a lower induction threshold and a graded response to inducer. B) Effect of the *lacO1* mutation on the maximum *lac* expression level. Mean P_lac_-GFP expression levels during growth in saturating levels of inducer (100 µM TMG) are shown for the ancestor, evolved clone G+L3-1, *lacO1* ^G11A^ single mutant and the G+L3-1 clone with *lacO1* reverted to the ancestral sequence (G+L3-1 *lacO1^anc^*). Standard error is shown, n = 4.

The *lacI* insertion/deletion and *lacO1* mutations are clearly good candidates to explain the evolved changes in *lac* operon regulation, but additional mutations may also be influential. To test the effect of the identified mutations on *lac* expression, we added the evolved *lacI* 4 bp deletion allele and the *lacO1* ^G11A^ mutation individually into the ancestral reporter strain. If these mutations play a major role in determining the evolved regulatory change, we expect these constructed strains to have inducer profiles similar to those of the evolved strains from which the mutations were isolated. Indeed, the *lacI* deletion recapitulated the inducer response profiles found in all constitutive clones, causing P_lac_-GFP expression levels to become independent of TMG concentration (Figure 6A, *lacI* ^ΔTGGC^ versus Figure 3A, Constitutive). Similarly, adding the *lacO1* ^G11A^ mutation into the ancestral background recreated the lower threshold/graded inducer response associated with evolved clones harboring mutations in *lacO1* (Figure 6A, *lacO1* ^G11A^ versus Figure 3A, Lower threshold). The TMG_½Max_ value for the *lacO1* ^G11A^ reconstructed strain was 4 µM, which is similar to the TMG_½Max_ of evolved clones with this mutation (G+L3-1, 8 µM; G+L5-1, 4 µM), demonstrating that the *lacO1* mutation is the primary cause of evolved changes in inducer sensitivity.

The *lacI* and *lacO1* mutations can explain many, but not all, of the *lac* regulation changes seen in the evolved strains. Specifically, neither mutation confers the increase in maximal expression that was seen in most evolved clones (Figure 6B, Figure 4B). To conclusively establish that additional evolved mutations impacted *lac* regulation in the G+L3-1 evolved clone, we replaced the *lacO1* mutation with the ancestral operator sequence. This strain maintained the ∼2-fold increase in maximum P_lac_-GFP expression level relative to the ancestor, indicating that mutations outside the canonical *lac* operon regulatory network contribute to evolved changes in *lac* regulation (Figure 6B). Whole genome sequencing of G+L3-1 found 6 additional mutations (in the genes or gene regions *rbsDACB*, *ECB_00822*, *fabF*, *sapF*, *mreB* and *malT*), none of which are in genes previously characterized as affecting *lac* operon expression.

### Fitness analysis of *lacI* and *lacO1* mutants

**Figure 7:**
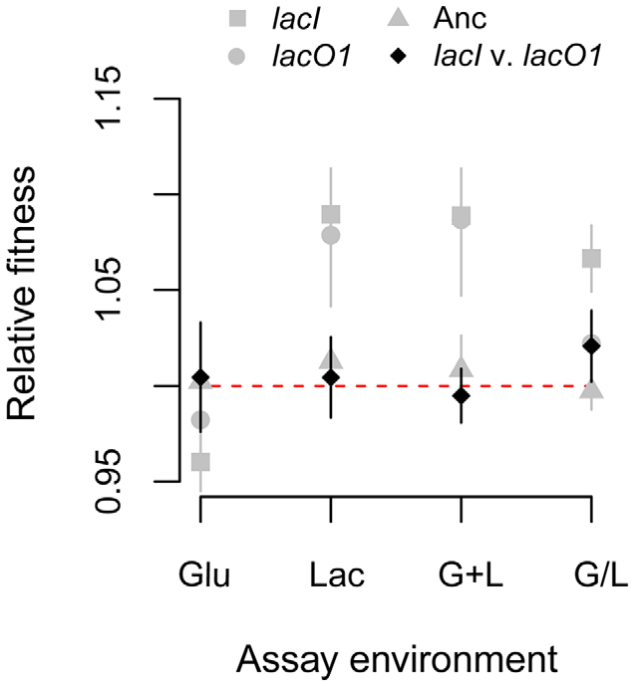
Effect of *lac* mutations on fitness. A) Fitness of *lacI* and *lacO1* mutants in the four evolution environments. Competitions were performed for *lacI* versus the ancestor, *lacO1* versus the ancestor and *lacI* versus *lacO1*. As a control, we included the ancestor competed against itself. The red dotted line indicates a relative fitness of 1 (no fitness difference). 95% confidence intervals are shown for each competition (n≥8).

That multiple *lacO1* and *lacI* mutations arose independently in replicate populations and affect a trait of relevance in the evolution environments suggests that they confer a selective advantage. Further, the presence of *lacO1* mutations exclusively in the G+L evolved populations suggests that they confer a greater advantage in this environment than do *lacI* mutations. To test these predictions we introduced the *lacI* and *lacO1* mutants individually into the ancestor and measured the fitness of these constructed strains relative to the ancestor in each of the four evolution environments. We found that *lacO1* and *lacI* mutations conferred a fitness benefit in all environments containing lactose (mean relative fitness effect and 2-tailed *t*-test: Lac environment. *lacI*: 8.9%, *P*<0.001; *lacO1*: 7.9%, *P*<0.001. G+L environment. *lacI*: 8.4%, *P*<0.001; *lacO1*: 8.0%, *P* = 0.001. G/L environment. *lacI*: 6.6%, *P*<0.001; *lacO1*: 2.2%, *P* = 0.02). By contrast, both mutations imposed a small fitness cost in the glucose environment, consistent with them not being observed in Glu populations (Glu environment: *lacI*: −4.0%, *P*<0.001; *lacO1*: −1.8%, *P* = 0.16) (Figure 7). Intriguingly, despite the *lacO1* mutations being significantly overrepresented among G+L populations, they did not confer a greater fitness advantage in this environment. Similarly, *lacI* mutations did not confer a greater advantage in Lac or G/L environments, where they were dominant. To address the possibility that some non-transitive interaction could complicate our indirect comparison of the relative fitness benefits of the two mutations, we also performed direct competitions between the two constructed strains. Again, we found that the fitness of the *lacI* mutant was not significantly different from that of the *lacO1* mutant in any environment (fitness of *lacI* relative to *lacO1*, 2-tailed *t*-test: Glu environment: 0.5%, *P* = 0.69; Lac environment: 0.4%, *P* = 0.60; G+L environment: −0.5%, *P* = 0.39; G/L environment: 2.1%, *P* = 0.05) (Figure 7).

### Physiological basis of the fitness advantage of *lacI* and *lacO1* mutants

**Figure 8:**
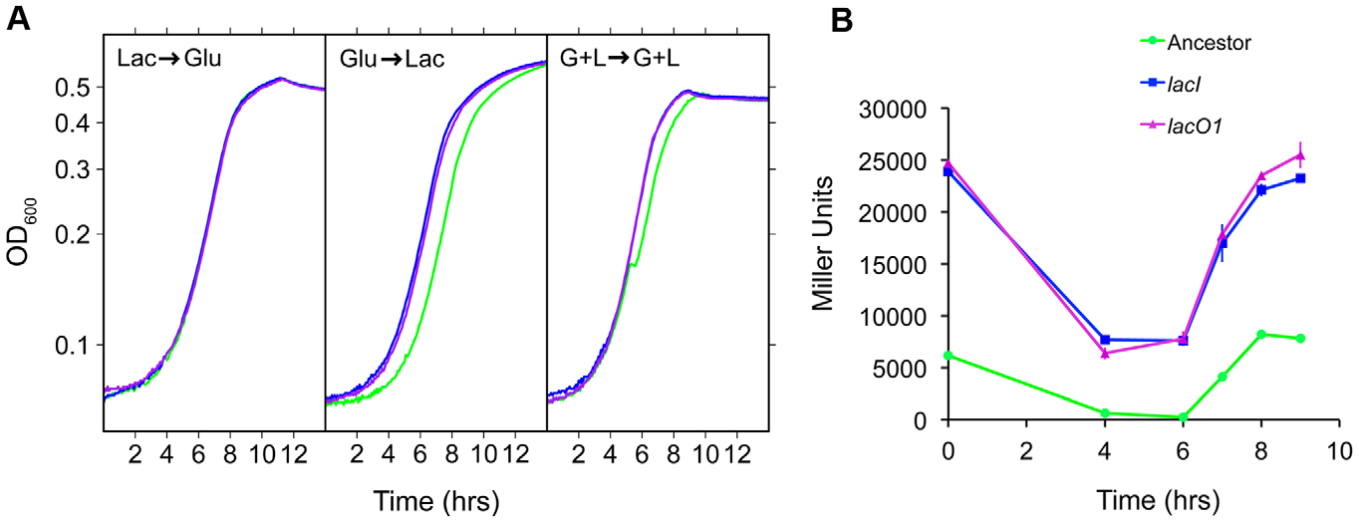
Physiological characterization of *lac* mutants. A) Growth curves for the ancestor (green), and reconstructed *lacI* (blue) and *lacO1* (purple) mutants. Conditions used were: Lac→Glu, Glu→Lac and G+L→G+L, where the sugars indicate pre-conditioning and measurement environments, respectively. These transitions correspond to those present in the G/L and G+L evolution environments. OD values are plotted on a log_10_ scaled axis. B) LacZ expression time course for the ancestor and the ancestor with *lacI* and *lacO1* mutations during growth in the G+L evolution environment. Values are the average of two independent replicates with standard deviation shown.

To examine the basis of the fitness effects conferred by the *lacI* and *lacO1* mutations, we quantified their effect on population growth dynamics, focusing on the transitions between glucose and lactose utilization that are encountered in the G+L and G/L environments (environments in which *lacO1* and *lacI* mutants predominated). We found that the *lacI* and *lacO1* mutations significantly decreased the lag phase, relative to the corresponding ancestral alleles, following a shift from growth on glucose to growth on lactose (a part of the G/L environment) (Figure 8A, Table 2). Furthermore, both *lacI* and *lacO1* mutations eliminated the diauxic lag phase measured for the ancestor when switching from glucose to lactose utilization in the G+L environment. Neither mutation had a significant effect on lag time following a shift from growth in lactose to glucose, indicating that the change in lag time was specific to lactose utilization. The maximum growth rate constant (μMax) for *lacI* and *lacO1* mutants was not significantly different from that of the ancestor, except during growth on lactose in the G+L environment (Figure 8A, Table 2). In this case, both *lacI* and *lacO1* mutants had significantly higher growth rates than the ancestor. In agreement with fitness measurements, strains containing the *lacI* and *lacO1* mutations show no significant differences in the length of lag phases or maximum growth rates in any of the environments tested. To decrease experimental noise in these experiments, we used higher sugar concentrations than present in the evolution experiment. Analysis of growth dynamics using the exact evolution environments yielded qualitatively similar results (Figure S3).

**Table 2:** Growth parameters for ancestor, *lacI* and *lacO1* mutants.

	Lac→Glu		Glu→Lac		G+L→G+L			
Strain	Lag (hrs)	μMax (hrs^−1^)	Lag (hrs)	μMax (hrs^−1^)	Lag-1 (hrs)	μMax (hrs^−1^)	Lag-2 (hrs)	μMax (hrs^−1^)#
Ancestor	3.85±0.10	0.474±0.006	4.84±0.09	0.470±0.011	3.18±0.04	0.370±0.013	0.47±0.04	0.461±0.006
*lacI*	3.83±0.09	0.468±0.007	3.59±0.07**	0.484±0.009	3.12±0.09	N/A	none	0.495±0.009*
*lacO1*	3.79±0.09	0.459±0.006	3.78±0.07**	0.478±0.008	3.10±0.10	N/A	none	0.496±0.008*

*Significantly different than ancestor, *P*<0.01 (two-tailed *t*-test, df = 10).

**Significantly different than ancestor, *P*<0.001 (two-tailed *t*-test, df = 10).

# μMax for *lacI* and *lacO1* is defined in an OD_600_ range that is most comparable with the OD_600_ range used to define μMax for the second exponential growth phase of the ancestor (μMax-2).

In contrast to the *lacI* mutant, the *lacO1* mutant can repress *lac* expression to some degree (Figure 2, Figure 3A). To examine how this difference in regulation translates to the evolution environment, we measured LacZ activity of the ancestor and the *lacI* and *lacO1* mutants in the G+L environment (Figure 8B). The ancestor shows the anticipated LacZ expression profile, with activity decreasing 97% during growth on glucose and increasing back up to the initial level of activity after switching from growth on glucose to lactose. Interestingly, *lacI* and *lacO1* mutants showed indistinguishable LacZ activity profiles, with LacZ activity much higher than the ancestor at all time points during growth in G+L medium. These results suggest that the relatively low levels of lactose present in the G+L environment induce the *lacO1* mutant *lac* operon, even in the presence of glucose concentrations sufficient to prevent induction of the ancestral *lac* operon. Analysis of the steady state levels of *lac* expression during growth in DM+Glu (2 mg/mL) with and without lactose (87.5 µg/mL) supports this conclusion, with repression of *lac* expression only occurring in the absence of lactose (Figure S4). In summary, in the ancestral background, *lacO1* and *lacI* mutations are indistinguishable with respect to their effect on fitness, growth dynamics and *lac* expression dynamics in the G+L environment.

### Genetic interactions with *lac* mutations and their effect on fitness

**Figure 9:**
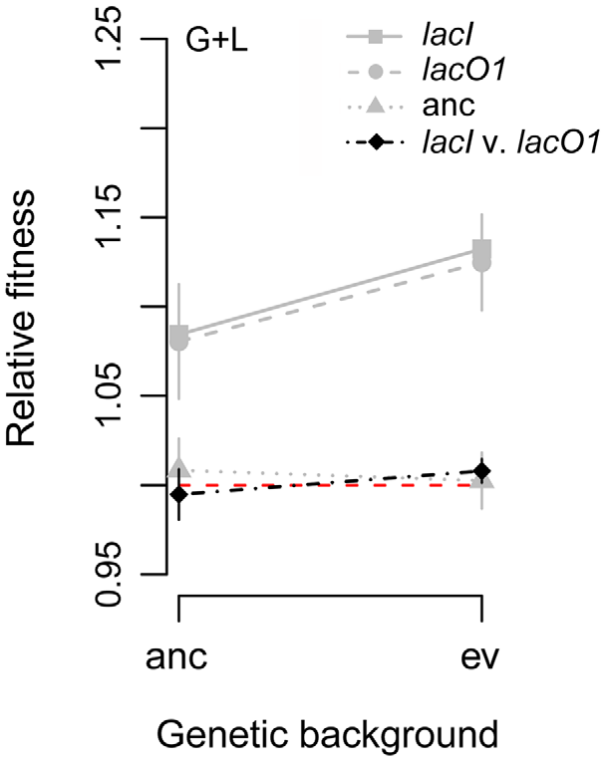
Fitness effect of *lacI* and *lacO1* mutations depend on genetic background. The fitness effect of *lacO1* and *lacI* mutations were measured in the ancestor and the G+L3-1 evolved genetic backgrounds when competed in the G+L environment. Gray points indicate fitness effect in competitions against the corresponding progenitor strains that do not have the added *lac* mutation; black points indicate fitness of *lacI* and *lacO1* mutations competed directly against each other. Lines connect competitions of the same type but in different genetic backgrounds.

A possible explanation for the success of *lacO1* mutations in the G+L populations despite them not conferring any advantage relative to more frequent *lacI* mutations is that they interact synergistically with other mutations that fixed during the evolution of these populations [37]. To test this, we compared the fitness advantage conferred by *lacO1* and *lacI* alleles in the ancestral background relative to the advantage they confer in the genetic background of evolved clone G+L3-1, which substituted a mutation in *lacO1* during evolution in the G+L environment (Figure 5). If epistasis was important in selection of *lacO1* alleles in the G+L environment, we predicted that the fitness advantage conferred by the *lacO1* mutation would be significantly larger in the background of this evolved clone than in the ancestral background, and that this positive effect will be less pronounced for the *lacI* mutation. The *lacO1* mutation did confer a bigger benefit in the evolved background (two tailed *t*-test, fitness in evolved background minus fitness in ancestral background = 4.4%, *P* = 0.03) (Figure 9). However, a similar effect was seen for the *lacI* mutation (two tailed *t*-test, fitness in evolved minus fitness in ancestral background = 4.8%, *P*<0.001) and there was no significant difference in the fitness conferred by the *lacI* and *lacO1* mutations in the evolved background when they were directly competed against one another (*lacI* versus *lacO1* in evolved background, relative fitness difference = 1.3%, *P* = 0.07). Therefore, epistatic interactions increase the fitness effect of both the *lacI* and *lacO1* mutations in the evolved background, but do not explain the enrichment of *lacO1* mutations in populations evolved in the G+L environment.

### Alternative explanations of the occurrence of *lacO1* mutations

In the absence of a measurable difference in the fitness conferred by *lacI* and *lacO1* mutations, what could explain our finding that *lacOI* mutations only occurred in populations evolved in the G+L environment? If the *lacI* and *lacO1* mutations occurred with equal probability, the distribution of mutation types over selection environments we observed is unlikely to have occurred by chance (Fisher's exact test omitting the polymorphic G+L population, *P* = 0.002). In fact, our observations are even more unlikely than this test implies because the *lacO1* mutation will almost certainly occur at a much lower rate than the *lacI* mutation. The frequency of *lacI* null mutations in the *E. coli* strain used in this experiment is ∼3×10^−6^ per generation (Hana Noh and TFC unpub. obs.), which is in good agreement with a previous measurement for *E. coli* K12 [31]. By contrast, we conservatively estimate the *lacO1* mutation frequency to be <3×10^−9^ per generation (Materials and Methods). Without some unique advantage, it is difficult to see how *lacO1* mutations could reach high frequency in any population, let alone predominate as in the G+L populations. The ∼1000 fold difference in mutation frequency does, however, provide an explanation for the absence of *lacO1* mutations in Lac or G/L environments.

We considered the possibility that cross-contamination could be responsible for the occurrence of identical *lacO1* mutations in two of the G+L populations, which would reduce the number of independent *lacO1* populations to three. This is unlikely since replicate populations were not propagated in adjacent wells, and no evidence for cross contamination was found during the course of the experiment (see Materials and Methods). Furthermore, the environmental association remains significant even if only unique *lacO1* mutations are considered (Fisher's exact test omitting one polymorphic population and populations with non-unique *lacO1* mutations, *P* = 0.006).

Finally, it is possible that the statistical association between the *lacO1* mutation and the G+L evolution environment, despite its high significance, is nevertheless spurious. In this case, repeat evolutionary experiments of the ancestor in the same G+L and G/L environments (where selection of *lacI* mutations was most consistent) would not be expected to lead to significant mutation-environment association. To test this, we began 12 ‘replay’ populations in each of the G+L and G/L environments, founding each population with the same ancestor as used in the original experiment. Every 100 generations, we examined the frequency of *lacI* and *lacO1* mutations in each population using LacZ indicator plates and by sequencing select clones. Although *lacO1* mutants were detected in the majority of G+L replay populations, their frequencies never rose above 4% in any one population (Table S1). In contrast, *lacI* mutants rose to high frequency, accounting for >98% of clones in all populations by 400 generations. Replay experiments in the G/L environment followed a similar trend, although *lacO1* mutations were detected in only 2 of the 12 populations over the course of the experiment (Table S2). In summary, despite being highly improbable, the failure of *lacO1* mutations to establish in our replay experiments suggests that their enrichment over competing *lacI* mutations in the original G+L populations may have occurred by chance.

## Discussion

We sought to test whether evolution in environments that differed only in the availability and presentation of lactose selected for changes in the regulation of the *lac* utilization network. Our analysis of inducer response profiles found three broad classes of *lac* regulation change among evolved clones. Two of these classes, constitutive expression and a lower threshold/graded inducer response, represent substantial changes from ancestral regulation and were observed only in populations evolved in environments containing lactose. Sequencing of *lac* regulatory regions in evolved clones uncovered mutations in the *lac* repressor (*lacI*) and the primary lac operator (*lacO1*) that correlated with the constitutive and the lower threshold/graded inducer response, respectively. Addition of these mutations to the ancestor demonstrated that they explained many, but not all, of the broad scale changes in regulation we observed and that, by themselves, they can confer fitness benefits in environments containing lactose. These fitness benefits were relatively large, representing 20%, 27% and 28% of the total mean fitness improvement in the Lac (*lacI* mutation), G+L (*lacO1* mutation) and G/L (*lacI* mutation) evolved populations, respectively [13]. Together these results indicate that regulatory changes were common, complex — occurring both within and outside of the recognized *lac* regulatory elements — and adaptive.

Extensive previous study of *lac* operon regulation offers the opportunity to connect the genetic and phenotypic changes we observed. Twenty-one of the 22 *lacI* mutants we identified mapped to a mutational hotspot within *lacI* [30], [31]. These mutations generate a frameshift in the coding sequence that results in expression of a nonfunctional repressor, which provides a good explanation for the complete loss of negative regulation we observed in *lacI* mutants. Similarly, the three *lacO1* mutations we identified in the G+L evolved populations have been reported as reducing the binding affinity of LacI for this binding site [33]–[35]. Consistent with the mutations reducing, but not completely preventing, LacI binding, strains containing *lacO1* mutations are able to repress the *lac* operon, but are induced at much lower TMG concentrations than the ancestor. *lacO1* mutations also conferred a graded response to increasing inducer concentration, which contrasted with the canonical bimodal response of the ancestor. The same regulatory outcome was demonstrated by Ozbudak et al. (2004), who found that decreasing the effective concentration of LacI by providing extra copies of *lacO1* binding sites resulted in a graded unimodal induction of the *lac* operon [19]. The similarity in regulatory changes suggests that the graded induction we observe is a consequence of decreased LacI-*lacO1* affinity, reducing the effective concentration of LacI repressor. More generally, our results support the concept that small changes in the activity of cis-regulators have the potential to transform the output of a regulatory network between binary and graded responses [38].

Both *lacI* and *lacO1* mutations were shown to confer significant fitness benefits in the three lactose containing evolution environments (Lac, G+L and G/L). Analysis of the growth dynamics of strains containing only these mutations indicated that a large part of this benefit is due to a reduction in lag phase when switching from glucose to lactose utilization. Interestingly, when added to the ancestor, both *lacO1* and *lacI* mutations abolished the diauxic lag that separates glucose and lactose growth phases during growth in the G+L environment. This phenomenon is well documented for *lacI* mutants [39], but to the best of our knowledge has not been demonstrated for *lacO1* mutants. In the case of *lacI* mutants, constitutive expression of the *lac* operon primes the cell for utilization of lactose as soon as glucose resources are exhausted. In contrast to *lacI* mutants, *lacO1* mutants are still capable of repressing *lac* expression in the absence of inducer. However, when grown in media containing both glucose and lactose the *lacO1* mutation essentially phenocopies a *lacI* mutant, causing constitutive *lac* expression. Evidently glucose-mediated blockage of lactose import through LacY (inducer exclusion) is insufficient to prevent lactose accumulating in cells to a concentration sufficient to allow *lac* operon induction in *lacO1* mutants [39], [40].

The loss of *lac* repression in lactose (3/6), but not glucose (0/6), evolved populations is consistent with the ‘use it or lose it’ hypothesis [23], [24]. This hypothesis proposes that negative regulation will be maintained during evolution in environments in which gene products, in this case the LacI repressor, are used because mutants that lose the repressor will needlessly express the *lac* operon and be selected against. If a repressor is seldom used, as in the lactose evolution environment, loss of function mutations will not be effectively selected against and can fix through genetic drift. However, in its simplest form, this mutation accumulation mechanism does not capture the dynamics of the regulatory changes we see. First, loss of the *lacI* repressor actually confers a benefit during growth on lactose, so that underlying mutations will increase in frequency faster than expected if they were neutral. Second, repressor mutations also occurred in environments where glucose was just as common as lactose. Analysis of growth curves suggest a mechanism for this; *lac* repressor mutants were able to quickly begin growth following a switch from glucose to lactose. Fitness measurements indicated that this advantage outweighed the cost of unnecessary *lac* expression during growth in glucose.

Given the large benefit conferred by *lacI* mutations in the Lac environment, it is interesting that only three of the six Lac populations were enriched for *lacI* mutations. We identify two possible explanations for this observation. First, clonal interference may have resulted in *lacI* mutations being outcompeted by higher effect beneficial mutations. Second, populations that did not enrich *lacI* mutations may have fixed alternative mutations that genetically interact with *lacI* mutations to reduce their fitness benefit. To distinguish between these possibilities, we are continuing the evolution experiment and tracking the frequency of *lacI* mutations in the Lac populations. In addition, we are examining the fitness benefit conferred by *lacI* mutations when introduced into clones from Lac evolved populations that did not fix *lacI*.

We can explain why *lac* mutations occurred only in lactose containing selective environments. A second layer of environment specificity is less clear; why do *lacO1* mutations only reach high frequency in the G+L environment? The *lacI* and *lacO1* mutations had no differential effect on fitness in either the ancestor or an evolved background and conferred indistinguishable growth dynamics in all evolution environments. Without a selective advantage over *lacI* mutations it is difficult to understand how *lacO1* mutants were fixed in 4 of 6 G+L populations, especially considering that the rate of *lacI* mutations is likely on the order of 1000-fold greater than for *lacO1* mutations. In the absence of a plausible mechanism to explain enrichment of *lacO1* mutations in the G+L environment, we investigated whether environment-specific selection of *lacO1* mutants was reproducible. This was not the case, with all 12 of the independent replay populations selected in G+L eventually fixing (>98%) *lacI* mutations. It remains formally possible that subtle differences in media or experimental conditions during competition assays or the replay evolution experiments could affect the fitness advantage experienced by *lacO1* mutants in focal G+L evolved populations. However, taken at face value, the different outcome between replay and primary populations suggests that, notwithstanding mutation rate differences and the strong statistical association between environment and mutation type, the enrichment of *lacO1* mutations over *lacI* mutations in the G+L environment might have occurred by chance.

Models incorporating interactions between key regulatory elements can successfully predict aspects of *lac* operon regulation [18], [19]. Nevertheless, recent studies demonstrate that regulation of the *lac* operon is evolutionarily plastic, such that interactions can arise or be altered to fine tune regulation and better fit *E. coli* to its environment [11], [14], [41]. By characterizing changes in regulation without *a priori* assumptions as to the nature of regulatory changes or the mutations causing them, we were able to identify evolved clones with changes in *lac* regulation that are likely due to novel interactions with mutations in genes outside of the canonical *lac* operon. Two results support this conclusion. First, we identified numerous clones with maximal steady state expression levels of the *lac* operon that were higher than the ancestor. Further examination of evolved clone G+L3-1 indicated that the increase in maximal *lac* expression level could not be explained by the *lacO1* mutation present in this clone. Second, the fitness benefit conferred by the *lacO1* mutation in this same evolved clone was significantly greater than in the ancestor, indicating that one or more evolved mutations interact with the *lacO1* mutation to determine its effect on fitness. Whole genome sequencing of G+L3-1 identified six additional mutations, however, none of these mutations mapped to the *lac* operon or genes known to directly impact CRP-cAMP activity. It seems likely, therefore, that one or more mutations in the G+L3-1 clone have directly or indirectly led to new regulatory control of the *lac* operon.

Is there an optimal level of *lac* expression in each of the three lactose environments? Dekkel and Alon (2005) found that, in the presence of a gratuitous inducer, *lac* operon expression evolved to a level predicted on the basis of a cost-benefit analysis, dependent on the concentration of lactose in the selection environment [14]. Our results support the idea that maximal expression level is a plastic feature of the *lac* operon and can be tuned to best fit the environment. At this time, however, we do not know the genetic or molecular basis for the widespread increase in maximum *lac* expression observed in many evolved clones. Possible ‘local’ explanations include: increases in the level of cAMP, mutations in the *lacZYA* genes that affect mRNA stability, or changes in DNA supercoiling that increase *lac* operon transcription. It is also possible that changes in *lac* maximum expression reflect an alteration in some global process. For example, changes in the function or concentration of ribosomes could affect expression of all genes. In future work we aim to identify the evolved mutations that are responsible for changes in maximum *lac* expression and then construct strains that will allow us to test the adaptive value of different expression levels as well as probe the underlying molecular mechanisms.

An additional widespread change in *lac* regulation was that evolved clones displaying bimodal inducer responses tended to also have higher induction thresholds (TMG_½Max_) than the ancestor. This trend was not environment specific, occurring in clones isolated from Glu, Lac and G/L evolved populations. However, the parallel and large-scale increases in induction threshold observed for Glu-evolved clones suggests that this change in *lac* regulation is a direct or correlated response to adaptation. The mechanistic bases of increases in induction threshold are currently not understood, but could be the result of both direct and indirect mechanisms. For example, reduction in the activity and/or concentration of the permease LacY could increase the concentration of extracellular inducer required to achieve intracellular levels of inducer sufficient to inactivate LacI. In glucose evolved populations, changes in LacY activity may result from mutations in the PTS system that optimize glucose transport but lead to elevated levels of unphosphorylated EIIA^glc^, which is a known inhibitor of LacY activity [40]. Alternatively, higher growth rates of evolved strains will also tend to decrease the steady state intracellular concentration of inducer thereby increasing the external concentration required for induction of the *lac* operon. Further study will be required to discern between these and other hypotheses.

In summary, we have identified and characterized widespread changes in *lac* operon regulation that occurred during selection of replicate populations in different lactose containing environments. In our view, the most important aspects of our findings are how common these changes were and that they likely involve mutations both within and outside of the set of genes that are recognized as regulating the *lac* operon. Identification of these changes will provide a rare insight into how regulatory networks can be rewired in response to an environmental change.

## Materials and Methods

### Strains and growth conditions

The ancestral strains used for experimental evolution studies were *E. coli* B REL606 (ara−) and an otherwise isogenic ara+ derivative, REL607 [42]. For routine culturing, cells were grown in lysogeny broth (LB) medium [43]. Davis minimal (DM) medium was used for experimental evolution and subsequent analysis of evolved clones [42]. Sugars were added to base DM medium at the following concentrations to make single and mixed resource environments: glucose (Glu) 175 µg/mL, lactose (Lac) 210 µg/mL, and Glucose+Lactose (Glu+Lac) 87.5 µg/mL and 105 µg/mL, respectively. These concentrations were chosen to ensure that each environment supports approximately the same stationary phase density of bacteria (∼3.5×10^8^ cfu/mL) [13]. Strains were grown at 37°C unless otherwise stated. T medium contains 1% Bacto tryptone, 0.1% yeast extract and 0.5% sodium chloride. Antibiotics were used at the following concentrations: chloramphenicol (Cm), 20 µg/mL; kanamycin (Km), 35 µg/mL; streptomycin (Sm), 100 µg/mL.

### Qualitative characterization of *lac* operon regulation within evolved populations

Evolved populations were screened for qualitative changes to *lac* operon regulation using TGX medium, which consisted of T agar plates supplemented with 0.5% glucose and 30 µg/mL of the colorimetric LacZ substrate 5-bromo-4-chloro-3-indolyl-beta-D-galactopyranoside (X-Gal). On this medium, the degree of blue coloration correlates with the degree of LacZ activity and, therefore, *lac* operon expression. Clones representing the diversity in LacZ activity and colony morphology within each evolved population were recovered, scored and stored for future analysis. TGX plates enabled us to distinguish between the ancestor and clones with *lacO1* and *lacI* mutations (mutations that we subsequently identified in evolved populations), which appear white, faint blue and dark blue, respectively.

### Quantitative tools for analysis of changes to *lac* operon regulation

We developed a dual fluorescent reporter system that enabled us to independently monitor LacI and CRP activity with single cell resolution. In this system the native *lac* promoter (containing LacI binding sites O1 and O3), coupled with an optimized ribosomal binding site, drives expression of the fast maturing GFP derivative, GFPmut3.1 [44]. This P_lac_-GFP module was cloned into a mini-Tn*7* delivery vector, to make pTn*7*-P_lac_-GFP, and integrated into the chromosome in a site-specific manner [45].

Expression of GFP from the P_lac_-GFP reporter depends on both LacI and CRP activity. To isolate these effects we developed a second reporter to monitor the contribution of CRP to *lac* operon expression independent of LacI. To do this, we constructed a version of the *lac* promoter (P_lacO(-)_) with defined mutations in the O1 and O3 operators which have been shown to prevent binding of the repressor LacI while maintaining the integrity of the major binding site for CRP [46]. This synthetic promoter was used to drive expression of the red shifted fluorescent protein DsRed express2 [47]. This reporter was cloned into a stable low copy plasmid (∼5 copies per cell) to generate pRM102-3. Control experiments confirmed that the introduced mutations abolished LacI binding while retaining promoter response to cAMP dependent activation of CRP (Figure S5). This reporter system differs from a previously published system in three important ways [19]. First, the fluorescent signal is bright enough to allow analysis by flow cytometry. Second, the CRP reporter is a derivative of the *lac* promoter that has the LacI binding sites deleted, rather than an unrelated reporter subject to regulation by CRP. Third, DsRed express2 shows low cytotoxicity relative to other commonly used RFP's [47].

### Transformation of evolved clones with LacI and CRP reporters

The Tn*7*-P_lac_-GFP reporter was integrated into the chromosome of focal evolved clones by tri-parental mating with the MFDpir (pTn*7*-P_lac_-GFP) donor strain and the MFDpir (pTSN2) helper strain [48]. Strains were grown overnight in LB and washed once in LB before being resuspended in 1/10 volume LB. Recipient, donor and helper strains were mixed at a 4∶1∶1 ratio and incubated on an LB plate for 3 hrs. Transconjugants were selected on LB plates supplemented with Km and Sm. A PCR assay was used to confirm that the mini-Tn*7* had inserted into the single characterized chromosomal integration site [49]. Finally, all reporter strains were screened to ensure that they retained the LacZ phenotype that formed the basis of their initial selection from evolved populations.

### Inducer response profiles

Strains were inoculated from glycerol stocks into 500 µL LB media in 2 mL deep-well plates (Phenix Research) and grown overnight at 37°C on a microplate shaker at 750 rpm (Heidolph Titramax 1000). After overnight growth, cultures were diluted 1∶1000 into 500 µL DM+0.4% glycerol and incubated for a further 24 hrs at 37°C. To determine the inducer response of each strain, overnight cultures were diluted 1∶1000 into separate wells of a 96-well plate containing DM+0.4% glycerol supplemented with TMG at concentrations ranging from 0 to 100 µM and incubated at 37°C for a further 15–18 hrs. This time period encompassed approximate steady state reporter expression in ancestral and evolved clones (Figure S6). TMG induces the *lac* operon by binding and inactivating the LacI repressor. Unlike the natural inducer, allolactose, TMG is metabolically stable, which is advantageous for quantitative studies because it allows accurate control of TMG concentrations through the course of the experiment. Importantly, import of TMG into the cell is dependent on the lactose permease LacY, which is not true of other commonly used synthetic inducers such as Isopropyl β-D-1-thiogalactopyranoside (IPTG).

Flow cytometry was performed with a FACScalibur (BD Biosciences) equipped with a high throughput sampler. PMT voltages for the flow cytometer were set as follows: SSC-H - E02, FSC-H 580 V, FL1-H 800 V and FL2-H 800 V. The threshold was set at 250 on the SSC-H channel. For each expression assay, a total of 25,000 events were captured at a rate of 1000–3000 events/s. Data was acquired in log mode with no hardware compensation. We examined day-to-day reproducibility by measuring ancestral inducer response curves on 5 separate days using independent cultures (Figure S7). The level and distribution of P_lac_-GFP expression in response to TMG was similar between replicates, indicating that our protocol for measuring inducer response profiles was robust.

### Manipulation and analysis of flow cytometry data

Routine analysis of the flow cytometry data and plotting of inducer response profiles was performed in R (version 2.12) using the Bioconductor packages FlowCore and FlowViz [50]–[52]. To control for cross talk between GFP and RFP reporter detection, a compensation matrix was calculated using the appropriate single reporter control strains and used to correct flow cytometry data post acquisition. To minimize detection noise and enrich for cells of similar size, all data was filtered with FlowCore's norm2Filter on the FSC-H and SSC-H channels using the default settings. This typically resulted in retention of 50–60% of all collected events.

For quantitative analysis of inducer responses, flow cytometry data were processed using Matlab (Mathworks, Inc.). An elliptical gate corresponding to a Mahalanobis distance of 0.5, centered at peak cell density in the FSC-SSC coordinates, was used to minimize the effects of varying cell size and granularity on the resulting fluorescence histograms. A custom bimodality detection algorithm (to be described elsewhere) was applied to determine the region of bimodality for each histogram. Inducer sensitivity was determined as the point where the population-mean of GFP expression was halfway between baseline and saturation.

### β-Gal assays

Assays were carried out as described by Zhang and Bremer (1995) with modifications [53]. Specifically, 1–2 mL of cell culture was pelleted and resuspended in 250 µL unsupplemented DM medium to remove any remaining lactose. Cell concentration was estimated by measuring absorbance at OD_600_ with a microplate reader (Tecan). Cells were permeabilized by mixing 20 µL of cell suspension with 80 µL of permeabilization solution (100 mM Na_2_HPO_4_, 20 mM KCl, 2 mM MgSO_4_, 0.8 mg/mL cetyl-trimethylammonium bromide (CTAB), 0.4 mg/mL sodium deoxycholate, 5 µL/mL β-mercaptoethonal). After incubation for 10 minutes at room temperature, measurement of LacZ activity was initiated by adding 150 µL of o-nitrophenyl-β-D-galactoside (ONPG, 4 mg/mL) and mixing. Yellow color development was stopped by addition of 250 µL 1 M sodium carbonate and the reaction time recorded. Samples were centrifuged to remove cell debris and absorbance at OD_420_ was measured for 200 µL of the supernatant. LacZ activity (Miller units) was calculated as (1000×OD_420_)/(volume (mL)×OD_600_×reaction time (min)).

### Growth analysis

Strains were inoculated into LB medium from freezer stocks, incubated overnight at 37°C and then diluted 1∶100 into DM medium supplemented with Glu, Lac or G+L at the concentrations used in the original evolution experiment. Strains were grown overnight and diluted 1∶100 into fresh DM media and incubated for a further 24 hrs to allow them to become physiologically adapted to their resource environment. To measure growth dynamics, a 1∶100 dilution of pre-conditioned culture was inoculated into 200 µL of DM supplemented with Glu, Lac or G+L in a clear 96-well plate. Concentration of sugars was either the same as in the evolution environments or to facilitate higher cell densities and correspondingly more precise OD measurements, were as follows: Glu, 0.2 mg/mL; Lac, 1.8 mg/mL; G+L, 0.2 mg/mL & 1.5 mg/mL, respectively. Incubation and optical density measurements were performed with a Bioscreen C plate reader (Oy Growth curves AB Ltd) at 37°C with continuous shaking and OD_600_ measured at 5 min intervals. The maximal growth rate constant (μMax) of each strain was calculated by linear regression of the plot of ln(OD_600_) versus time (hrs) using a sliding window of 10 data points. The steepest of these slopes was used to calculate μMax with units hrs^−1^. Lag time was calculated by extrapolating the μMax regression line to its intersection with OD_600_ = 0.06. Extrapolating to the initial density of individual growth curves would have been preferable, however, we found that these measurements were quite variable. To account for this we adopted the approach described by Friesen et al. (2004) where a constant reference density is used, assuming that starting biomass is similar for all strains [54]. For strains showing diauxic growth, we analyzed both growth phases separately to derive μMax-1 and μMax-2. The diauxic lag phase (lag-2) was calculated by determining the difference between the times when the regression lines for μMax-1 and μMax-2 intersect with the OD_600_ value coinciding with the end of the first growth phase.

### Promoter activity assays

Assays were performed as described by Kuhlman et al. (2007) [29]. Briefly, steady state levels of LacZ activity were measured for strains grown in DM+0.2% glucose supplemented with a saturating concentration of the gratuitous inducer IPTG (1 mM). β-Gal assays were performed as described above except that color development was followed over time by measuring absorbance at OD_420_ and linear regression used to fit a line of best slope to the plot of OD_420_ vs time (min). LacZ activity (Miller units) was calculated as (1000×slope)/(assay volume (mL)×OD_600_). Doubling rate was measured for each strain in the experimental conditions by linear regression of log_2_(OD_600_) plotted against time, with the steepest of these slopes designated as the maximum doubling rate (doublings/hr). Promoter activity was calculated as the product of LacZ activity (Miller units) and doubling rate (hrs^−1^). To confirm that the inducer concentration we used was sufficient to completely inactivate LacI, we also measured expression from a *lacI* null mutant. This mutation caused a similar expression increase as induction with 1 mM IPTG, indicating that this level of inducer was sufficient to fully induce the *lac* operon.

### Allele exchange and strain construction

Constructs and approaches used for the manipulation of each mutation were as follows. The P_lacO1_ and *araA*- mutations were introduced using a suicide plasmid approach that has been described previously [6]. Briefly, PCR products containing the relevant evolved alleles were separately cloned into pDS132 [55]. Resulting plasmids were introduced into recipients by conjugation and Cm^R^ cells (formed by chromosomal integration of the plasmid) were selected. Resistant clones were streaked onto LB+sucrose agar to select cells that lost the plasmid (which carries the *sacB* gene conferring susceptibility to killing by sucrose). These cells were then screened for the presence of the evolved alleles by a PCR-RFLP approach using the enzyme HaeIII (*araA-*) or on LacZ indicator medium (P_lacO1_). Putative allelic replacements of the evolved *ara- and lacO1* alleles were confirmed by sequencing.

The *lacI(-)* mutation was obtained by isolating spontaneous mutants of relevant strains that could grow on minimal media supplemented with P-Gal as the only carbon source and confirmed by sequencing [56]. We isolated independent *lacI(-)* mutants that had either insertion or deletion mutations in a previously identified mutational hotspot [57]. Preliminary experiments indicated that these mutant types had identical fitness in each of the environments used here. For this reason, we used only the deletion mutant in the experiments reported in Results.

### Fitness assays and analysis

The fitness of constructed strains was measured relative to the ancestor strain used to found the evolution experiment or directly to each other. Competing strains contained opposite Ara marker or *lac* regulation types, which allowed them to be distinguished on tetrazolium arabinose (TA) [42] or LB+X-Gal indicator medium, respectively. Before each fitness assay, competing strains were grown separately for one complete propagation cycle in the environment to be used in the assay so that they reached comparable cell densities and physiological states. Following this step, competitors were each diluted 1∶200 into the assay environment. A sample was taken immediately and plated on indicator plates to estimate the initial densities of the competing strains. At the end of the competition a further sample was plated to obtain the final density of each competitor. The fitness of the test strain relative to the reference strain was calculated as ln(N_T2_/N_T0_)/ln(N_R2_/N_R0_), where N_T0_ and N_R0_ represent the initial densities of the test and reference strains, respectively, and N_T2_ and N_R2_ represent their corresponding densities at the end of the competition with correction for the number of transfer cycles the competition occurred over. Competitions were generally carried out over two transfer cycles. All assays were carried out with at least four-fold replication unless reported otherwise in Results.

### Estimation of *lacO1* mutation frequency

To estimate the per generation frequency of loss of function *lacO1* mutations, we assume that mutation of the *lacO1* region is random and equally likely for each of the 21 bp that define *lacO1*. Using a mutation rate of 5×10^−10^ bp/generation [58] the probability of generating a single substitution in *lacO1* is ∼1×10^−8^ per generation. However, only a subset of these mutations will severely compromise LacI binding. A survey of the literature indicates that approximately 16 single base pair substitutions within *lacO1* have been reported to reduce the affinity of LacI by >90% and/or reduce the effective repression of LacZ expression >90% [33], [34]. Based on this, we conservatively estimate that a third of the possible 63 single base pair substitutions will severely compromise LacI binding, giving a mutation frequency of ∼3×10^−9^ per generation. This is likely an overestimate since only three *lacO1* alleles were selected during evolution, two of which were selected twice in independent populations, indicating that relatively few of the possible *lacO1* mutations may actually confer an advantage in the evolution environments. In addition, the *lacO1* alleles selected during evolution have been reported to reduce the affinity for LacI by >98%, further reinforcing the stringency of our operational criteria for estimating the number of possible *lacO1* mutations [33], [36].

## Supporting Information

Figure S1 CRP activity is independent of *lac* expression state in evolved clones. Inducer response histograms of the CRP-only reporter (P_lac(O-)_-RFP) for all evolved clones and the ancestor. In all cases, P_lac(O-)_-RFP expression is unimodal and not affected by LacI activity or *lac* operon expression state.
(TIF)

Figure S2 Effect of a *lacO1* mutation on *lac* operon expression. A) Inducer response profiles using P_lac_-GFP and P_lacO1_-GFP reporters in ancestral and G+L3-1 strains. B) Steady state LacZ activity for strains grown in DM+0.4% glycerol supplemented with a range of TMG concentrations.
(TIF)

Figure S3 Growth dynamics of *lacI* and *lacO1* mutants in evolution environments. A) Growth curves for the ancestor (green), and ancestor with *lacO1* (purple) and *lacI* (blue) mutations. Conditions used were: Lac→Glu, Glu→Lac and G+L→G+L, where the sugars indicate pre-conditioning and measurement environments, respectively. Concentration of sugars for both pre-conditioning and measurement environments matched those used in the evolution experiment. These transitions correspond to those present in the G/L and G+L environments. OD values are plotted on a log_10_ scaled axis. Growth curves show the same qualitative trends as described for growth analysis in environments with higher concentrations of sugars (Figure 8A, Table 2).
(TIF)

Figure S4 Effectiveness of inducer exclusion at repressing *lac* expression. Steady state P_lac_-GFP levels for ancestor, *lacI* and *lacO1* strains grown in DM+Glu (0.02–0.2%) supplemented with varying levels of lactose or the gratuitous inducer IPTG. Steady state conditions were obtained by starting cultures with small numbers of cells and back diluting cultures into pre-warmed fresh media when they reached an OD_600_ of ∼0.1, thereby keeping cells in exponential growth. After two propagation cycles GFP levels were measured with a flow cytometer. Under these growth conditions cells do not exhaust glucose levels and do not utilize lactose as a carbon source. As can be seen, *lacO1* mutants induce their *lac* operon in the presence of low external concentrations of lactose, even during growth on relatively high concentrations of glucose.
(TIF)

Figure S5 Efficacy of CRP-dependent reporter pRM102-3. A) Insensitivity of pRM102-3 to activity of LacI. Strains harboring both P_lac_-GFP and P_lacO(-)_-RFP reporters were grown in conditions with fully active LacI (no TMG) and fully inactive LacI (100 µM TMG). P_lac_-GFP responds to the changes in LacI activity whereas the P_lacO(-)_-RFP reporter is invariant with respect to inducer concentration. B) Dependence of pRM102-3 reporter on CRP and cAMP. Expression of the P_lacO(-)_-RFP reporter was measured in a *cya*, *cpdA*, *lacY* mutant over a range of concentrations of exogenous cAMP. This strain cannot make or degrade cAMP, ensuring that the reporter is responding to the external source of cAMP. As can be seen, RFP levels increase as a function of cAMP levels. To verify that the increases in expression were CRP dependent we looked at the response of the P_lacO(-)_-RFP reporter in a *crp* null mutant background. No increase in expression above background was seen.
(TIF)

Figure S6 Defining a steady state window for flow cytometry analysis. A) Inducer response profiles (P_lac_-GFP) for the ancestor measured at three different time points (14.5, 16.5 and 18.5 hours). Inducer response profiles are stable over this time range and for TMG concentrations with bimodal expression profiles the ratio of cells in the low and high expression states remains constant. B) Mean values for the P_lac_-GFP reporter over time for the ancestor and nine evolved clones grown in DM+Glycerol (0.4%) supplemented with 4 different TMG concentrations (0,1,10 & 100 µM), except the ancestor where TMG concentrations are 0,4,10 & 100 µM. Expression levels were relatively stable between 15–18 hours of growth, which we operationally defined as the pseudo-steady state window.
(TIF)

Figure S7 Reproducibility of inducer response measurements. A) Inducer response profiles for the ancestor (P_lac_-GFP) run on five independent days. Flow cytometry traces are overlaid at each TMG concentration. Numbers at the top of individual panels indicate the concentration of TMG (µM) present during population growth. B) Mean response of the ancestral inducer response profiles. Standard error is shown (n = 5) and varied from 1–8% of the mean value over the range of TMG concentrations tested, except at 20 µM TMG which showed slightly larger variation in mean RFU due to small differences in the proportion of cells in low and high expression states.
(TIF)

Table S1 Frequency of alleles (wt, *lacI*, and *lacO1*) within 12 replicate populations of *E. coli* B REL606 after propagation for 100, 200, 300, and 400 generations in the G+L environment.
(DOC)

Table S2 Frequency of alleles (wt, *lacI*, and *lacO1*) within 12 replicate populations of *E. coli* B REL606 after propagation for 100, 200, 300, and 400 generations in the G/L environment.
(DOC)
