# Supplementary figures and images for: Adaptive Evolution of the Lactose Utilization Network in Experimentally Evolved Populations of Escherichia coli
Source: PLoS Genet. 2012 Jan 12;8(1):e1002444. doi: 10.1371/journal.pgen.1002444 (PMC3257284; doi:10.1371/journal.pgen.1002444)

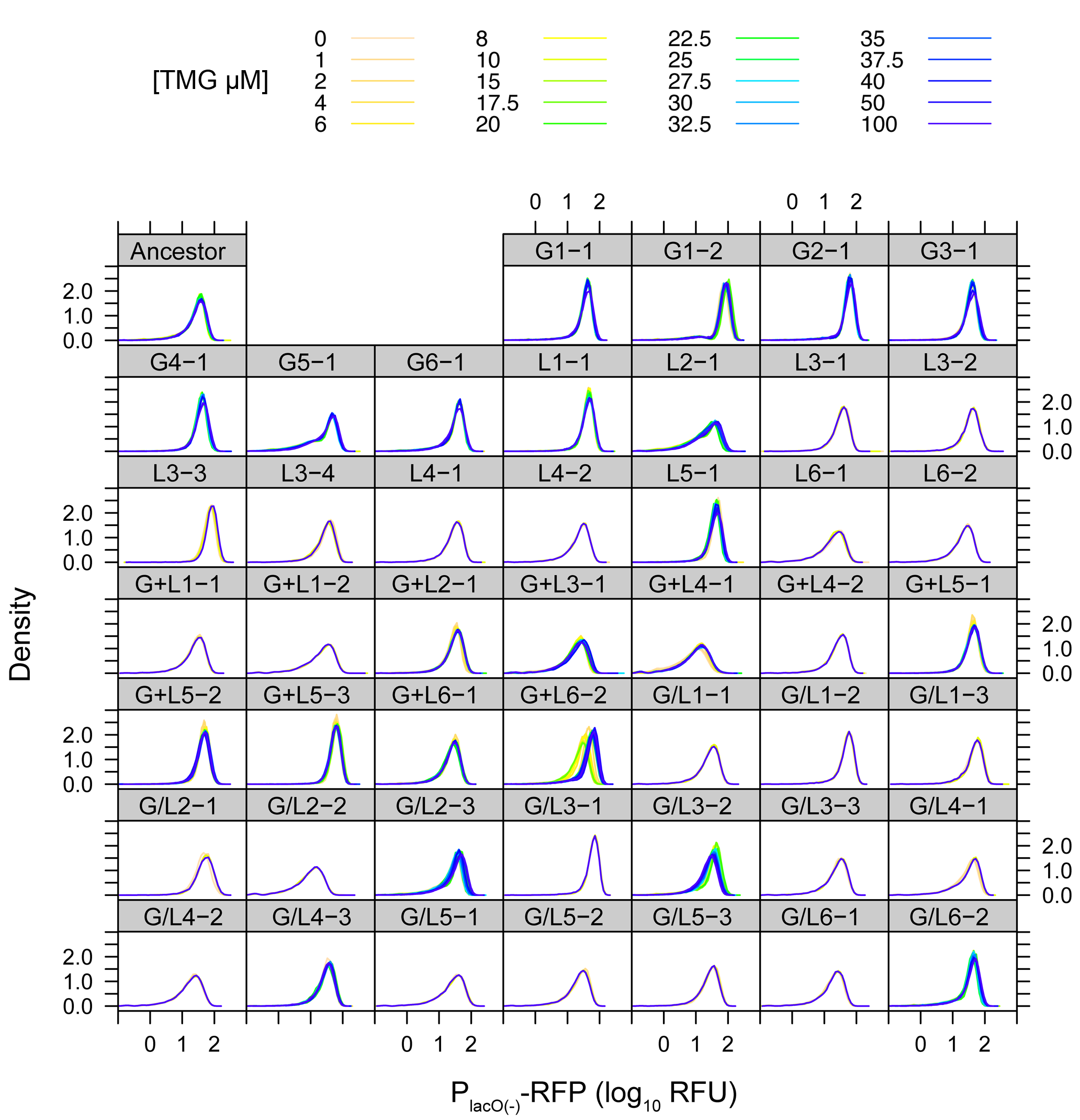

Supplement: Figure S1 — CRP activity is independent of lac expression state in evolved clones. Inducer response histograms of the CRP-only reporter (Plac(O-)-RFP) for all evolved clones and the ancestor. In all cases, Plac(O-)-RFP expression is unimodal and not affected by LacI activity or lac operon expression state. (TIF) [file pgen.1002444.s001.tif]

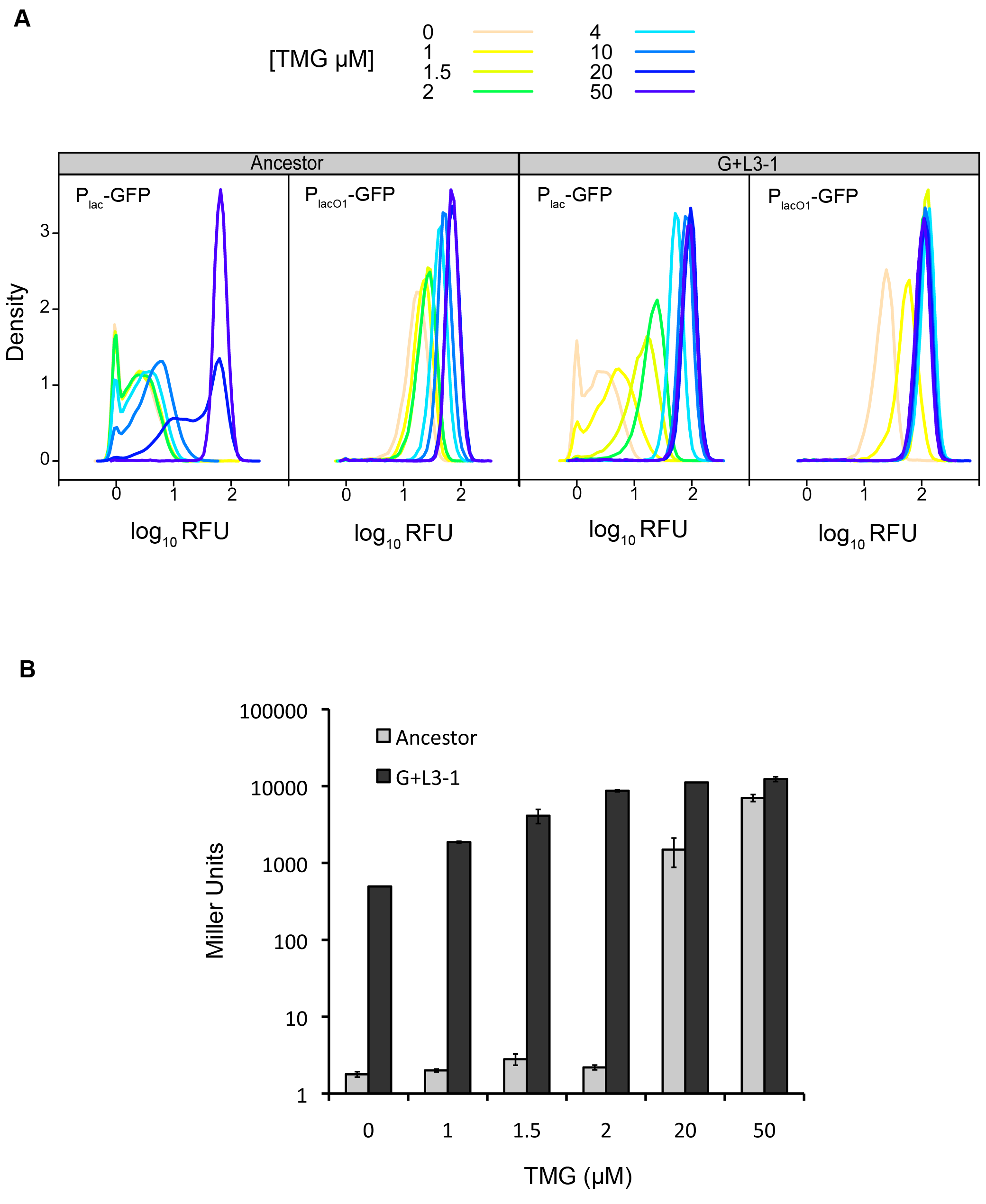

Supplement: Figure S2 — Effect of a lacO1 mutation on lac operon expression. A) Inducer response profiles using Plac-GFP and PlacO1-GFP reporters in ancestral and G+L3-1 strains. B) Steady state LacZ activity for strains grown in DM+0.4% glycerol supplemented with a range of TMG concentrations. (TIF) [file pgen.1002444.s002.tif]

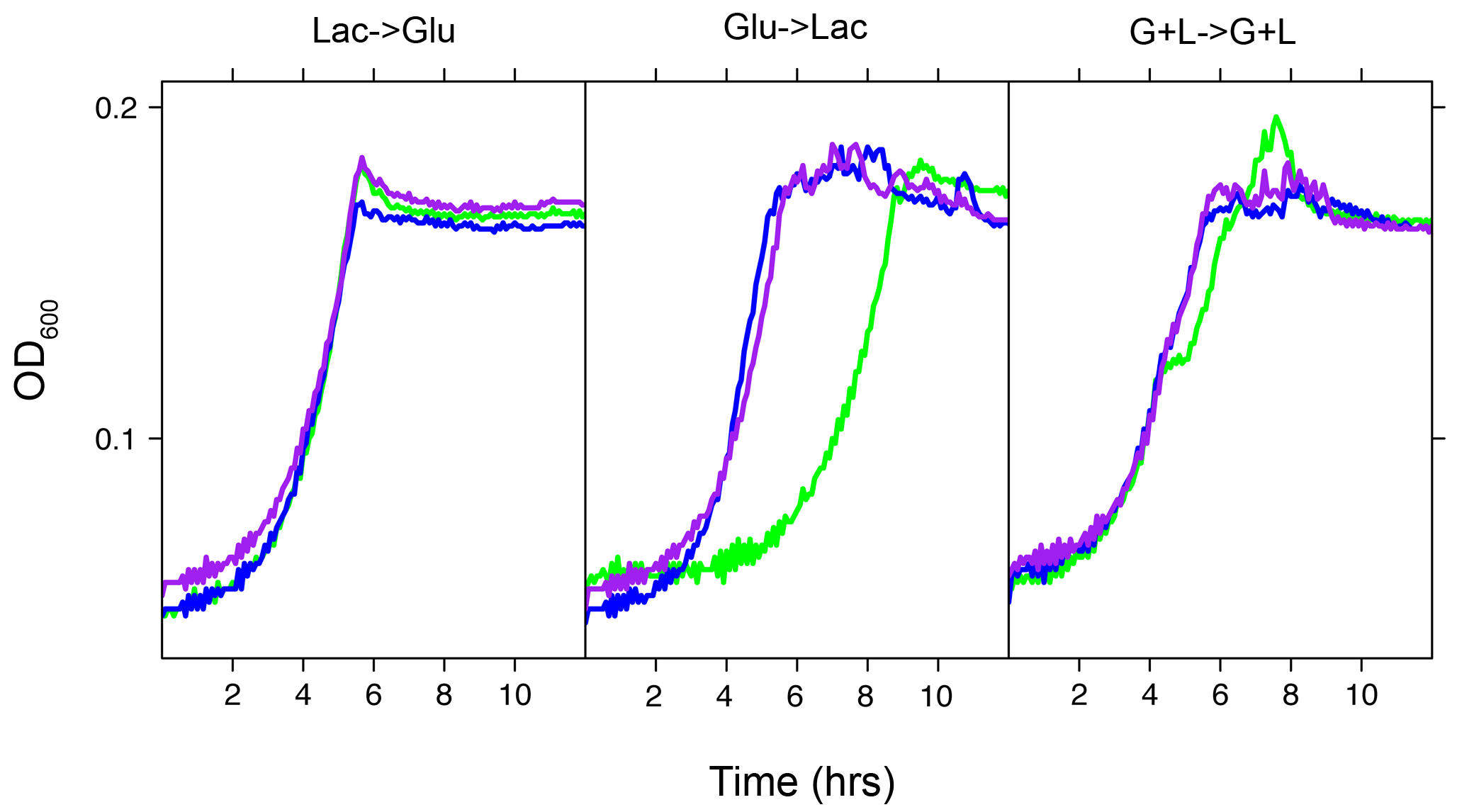

Supplement: Figure S3 — Growth dynamics of lacI and lacO1 mutants in evolution environments. A) Growth curves for the ancestor (green), and ancestor with lacO1 (purple) and lacI (blue) mutations. Conditions used were: Lac→Glu, Glu→Lac and G+L→G+L, where the sugars indicate pre-conditioning and measurement environments, respectively. Concentration of sugars for both pre-conditioning and measurement environments matched those used in the evolution experiment. These transitions correspond to those present in the G/L and G+L environments. OD values are plotted on a log10 scaled axis. Growth curves show the same qualitative trends as described for growth analysis in environments with higher concentrations of sugars (Figure 8A, Table 2). (TIF) [file pgen.1002444.s003.tif]

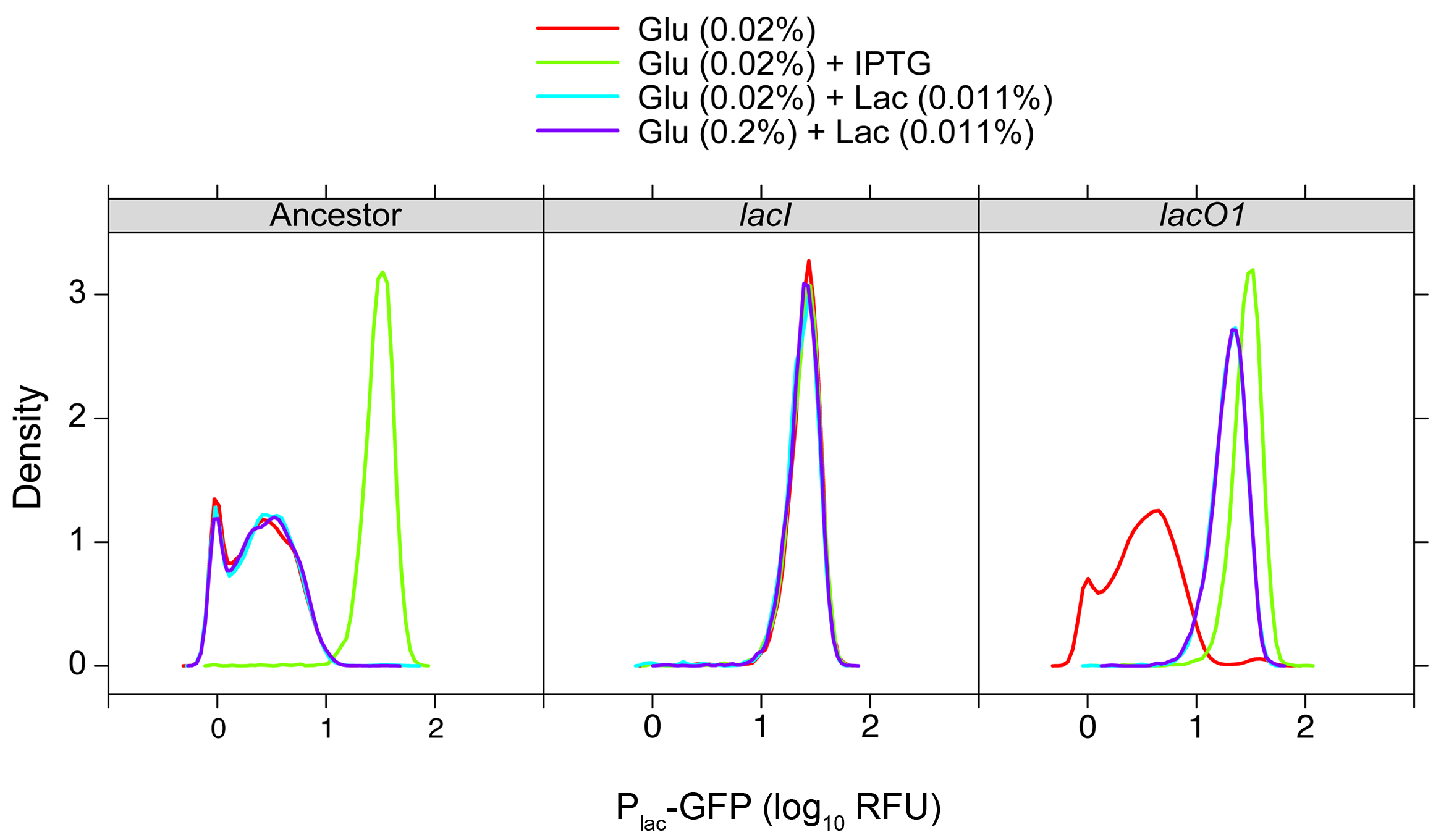

Supplement: Figure S4 — Effectiveness of inducer exclusion at repressing lac expression. Steady state Plac-GFP levels for ancestor, lacI and lacO1 strains grown in DM+Glu (0.02–0.2%) supplemented with varying levels of lactose or the gratuitous inducer IPTG. Steady state conditions were obtained by starting cultures with small numbers of cells and back diluting cultures into pre-warmed fresh media when they reached an OD600 of ∼0.1, thereby keeping cells in exponential growth. After two propagation cycles GFP levels were measured with a flow cytometer. Under these growth conditions cells do not exhaust glucose levels and do not utilize lactose as a carbon source. As can be seen, lacO1 mutants induce their lac operon in the presence of low external concentrations of lactose, even during growth on relatively high concentrations of glucose. (TIF) [file pgen.1002444.s004.tif]

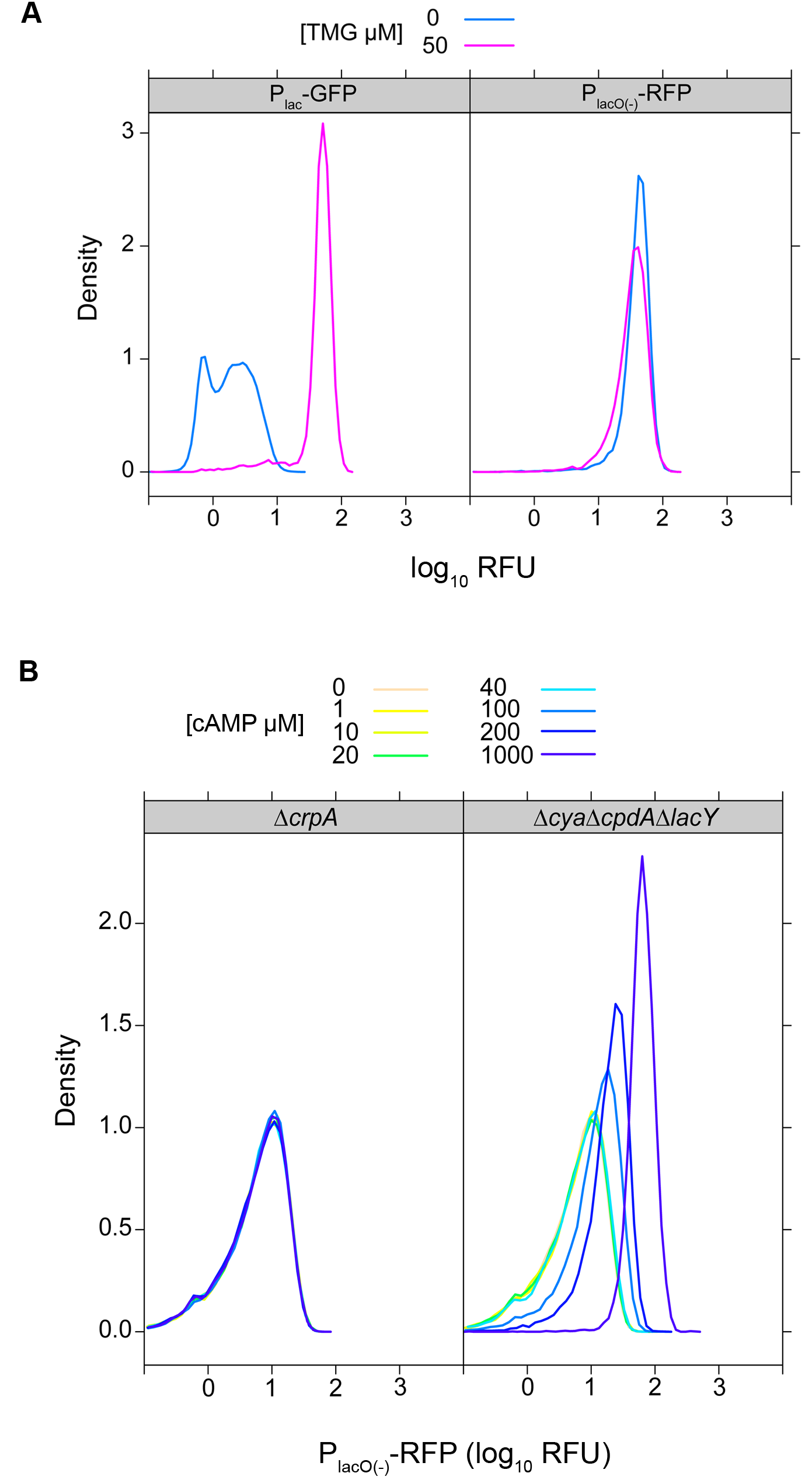

Supplement: Figure S5 — Efficacy of CRP-dependent reporter pRM102-3. A) Insensitivity of pRM102-3 to activity of LacI. Strains harboring both Plac-GFP and PlacO(-)-RFP reporters were grown in conditions with fully active LacI (no TMG) and fully inactive LacI (100 µM TMG). Plac-GFP responds to the changes in LacI activity whereas the PlacO(-)-RFP reporter is invariant with respect to inducer concentration. B) Dependence of pRM102-3 reporter on CRP and cAMP. Expression of the PlacO(-)-RFP reporter was measured in a cya, cpdA, lacY mutant over a range of concentrations of exogenous cAMP. This strain cannot make or degrade cAMP, ensuring that the reporter is responding to the external source of cAMP. As can be seen, RFP levels increase as a function of cAMP levels. To verify that the increases in expression were CRP dependent we looked at the response of the PlacO(-)-RFP reporter in a crp null mutant background. No increase in expression above background was seen. (TIF) [file pgen.1002444.s005.tif]

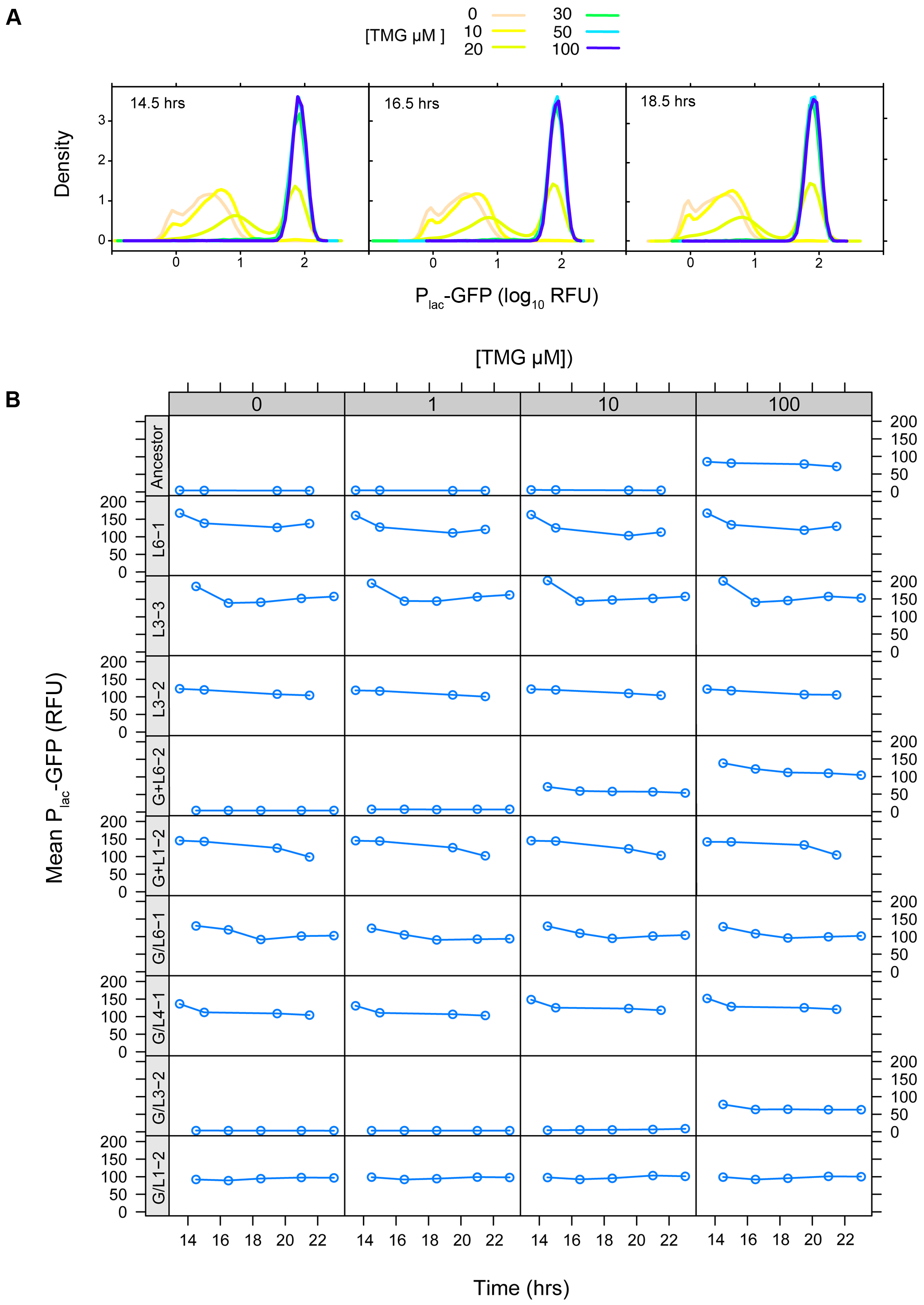

Supplement: Figure S6 — Defining a steady state window for flow cytometry analysis. A) Inducer response profiles (Plac-GFP) for the ancestor measured at three different time points (14.5, 16.5 and 18.5 hours). Inducer response profiles are stable over this time range and for TMG concentrations with bimodal expression profiles the ratio of cells in the low and high expression states remains constant. B) Mean values for the Plac-GFP reporter over time for the ancestor and nine evolved clones grown in DM+Glycerol (0.4%) supplemented with 4 different TMG concentrations (0,1,10 & 100 µM), except the ancestor where TMG concentrations are 0,4,10 & 100 µM. Expression levels were relatively stable between 15–18 hours of growth, which we operationally defined as the pseudo-steady state window. (TIF) [file pgen.1002444.s006.tif]

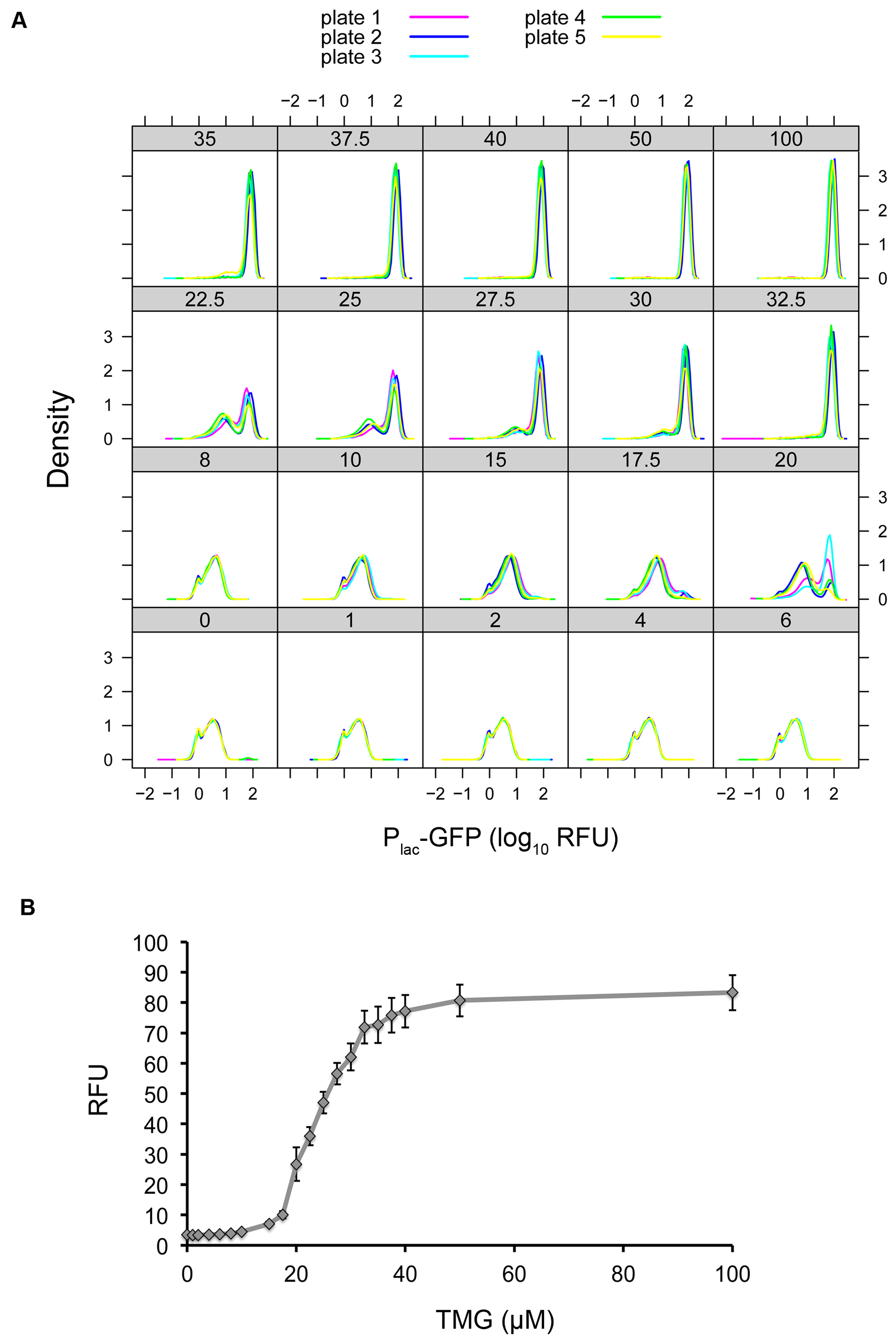

Supplement: Figure S7 — Reproducibility of inducer response measurements. A) Inducer response profiles for the ancestor (Plac-GFP) run on five independent days. Flow cytometry traces are overlaid at each TMG concentration. Numbers at the top of individual panels indicate the concentration of TMG (µM) present during population growth. B) Mean response of the ancestral inducer response profiles. Standard error is shown (n = 5) and varied from 1–8% of the mean value over the range of TMG concentrations tested, except at 20 µM TMG which showed slightly larger variation in mean RFU due to small differences in the proportion of cells in low and high expression states. (TIF) [file pgen.1002444.s007.tif]
