# Supplementary material for: Adaptive Evolution of the Lactose Utilization Network in Experimentally Evolved Populations of Escherichia coli
Source: PLoS Genet. 2012 Jan 12;8(1):e1002444. doi: 10.1371/journal.pgen.1002444 (PMC3257284; doi:10.1371/journal.pgen.1002444)
Supplement: Table S1 — Frequency of alleles (wt, lacI, and lacO1) within 12 replicate populations of E. coli B REL606 after propagation for 100, 200, 300, and 400 generations in the G+L environment. (DOC) [file pgen.1002444.s008.doc]

**Table S1.** Frequencies of *lac* mutants in G+L evolution replay populations

|  |  | **Replicate population** | | | | | | | | | | | |
| --- | --- | --- | --- | --- | --- | --- | --- | --- | --- | --- | --- | --- | --- |
| **Generation** | **Genotype (%)** | **1** | **2** | **3** | **4** | **5** | **6** | **7** | **8** | **9** | **10** | **11** | **12** |
| 100 | Anc | 94.2 | 67.9 | 76.2 | 53.4 | 58.1 | 99.4 | 10.4 | 60.3 | 80.5 | 59.8 | 73.0 | 52.8 |
|  | *lacI* | 5.6 | 30.3 | 23.0 | 46.2 | 41.9 | 0.3 | 89.2 | 38.3 | 17.2 | 39.9 | 27.0 | 47.2 |
|  | *lacO1* | 0.3 | 1.8 | 0.8 | 0.3 | 0.0 | 0.3 | 0.4 | 1.4 | 2.3 | 0.3 | 0.0 | 0.0 |
| 200 | Anc | 4.9 | 2.3 | 1.4 | 0.8 | 2.2 | 20.6 | 0.0 | 0.9 | 1.6 | 1.3 | 2.3 | 1.4 |
|  | *lacI* | 91.5 | 97.7 | 97.5 | 96.4 | 97.8 | 77.9 | 100 | 98.5 | 95.9 | 98.7 | 96.8 | 98.6 |
|  | *lacO1* | 3.7 | 0.0 | 1.1 | 2.8 | 0.0 | 1.4 | 0.0 | 0.6 | 2.5 | 0.0 | 1.0 | 0.0 |
| 300 | Anc | 0.0 | 0.3 | 0.0 | 0.3 | 0.0 | 38.8 | 0.3 | 0.6 | 1.4 | 0.8 | 0.9 | 0.6 |
|  | *lacI* | 99.2 | 99.4 | 100 | 99.4 | 100 | 58.8 | 99.7 | 98.7 | 98.6 | 99.2 | 98.7 | 99.2 |
|  | *lacO1* | 0.8 | 0.3 | 0.0 | 0.3 | 0.0 | 2.4 | 0.0 | 0.6 | 0.0 | 0.0 | 0.3 | 0.3 |
| 400 | Anc | 0.0 | 0.4 | 0.0 | 0.0 | 0.0 | 1.9 | 0.2 | 0.3 | 0.0 | 0.3 | 1.0 | 0.3 |
|  | *lacI* | 100 | 99.6 | 100 | 100 | 100 | 98.1 | 99.8 | 99.7 | 100 | 99.7 | 99.0 | 99.7 |
|  | *lacO1* | 0.0 | 0.0 | 0.0 | 0.0 | 0.0 | 0.0 | 0.0 | 0.0 | 0.0 | 0.0 | 0.0 | 0.0 |
