# Supplementary material for: Adaptive Evolution of the Lactose Utilization Network in Experimentally Evolved Populations of Escherichia coli
Source: PLoS Genet. 2012 Jan 12;8(1):e1002444. doi: 10.1371/journal.pgen.1002444 (PMC3257284; doi:10.1371/journal.pgen.1002444)
Supplement: Table S2 — Frequency of alleles (wt, lacI, and lacO1) within 12 replicate populations of E. coli B REL606 after propagation for 100, 200, 300, and 400 generations in the G/L environment. (DOC) [file pgen.1002444.s009.doc]

**Table S2.** Frequencies of *lac* mutants in G/L evolution replay populations.

|  |  | **Replicate population** | | | | | | | | | | | |
| --- | --- | --- | --- | --- | --- | --- | --- | --- | --- | --- | --- | --- | --- |
| **Generation** | **Genotype (%)** | **1** | **2** | **3** | **4** | **5** | **6** | **7** | **8** | **9** | **10** | **11** | **12** |
| 100 | Anc | 100 | 100 | 98.7 | 97.2 | 98.4 | 99.5 | 98.5 | 100 | 99.2 | 99.5 | 98.6 | 100 |
|  | *lacI* | 0.0 | 0.0 | 1.3 | 2.8 | 1.6 | 0.5 | 1.5 | 0.0 | 0.8 | 0.5 | 1.4 | 0.0 |
|  | *lacO1* | 0.0 | 0.0 | 0.0 | 0.0 | 0.0 | 0.0 | 0.0 | 0.0 | 0.0 | 0.0 | 0.0 | 0.0 |
| 200 | Anc | 72.7 | 92.9 | 24.2 | 61.7 | 19.7 | 32.8 | 62.9 | 75.2 | 34.0 | 62.8 | 76.0 | 98.2 |
|  | *lacI* | 27.3 | 5.2 | 4.7 | 38.3 | 80.3 | 67.2 | 37.1 | 24.8 | 66.0 | 37.2 | 24.0 | 1.8 |
|  | *lacO1* | 0.0 | 1.9 | 71.1 | 0.0 | 0.0 | 0.0 | 0.0 | 0.0 | 0.0 | 0.0 | 0.0 | 0.0 |
| 300 | Anc | 27.6 | 11.0 | 16.9 | 15.0 | 1.9 | 22.9 | 14.6 | 9.2 | 6.3 | 7.6 | 27.5 | 63.5 |
|  | *lacI* | 72.4 | 87.6 | 55.2 | 85.0 | 98.1 | 77.1 | 85.4 | 90.8 | 93.7 | 92.4 | 72.5 | 36.0 |
|  | *lacO1* | 0.0 | 1.4 | 27.9 | 0.0 | 0.0 | 0.0 | 0.0 | 0.0 | 0.0 | 0.0 | 0.0 | 0.5 |
| 400 | Anc | n/c | 0.0 | 44.1 | 0.9 | 0.0 | 1.3 | 1.5 | 0.4 | 0.6 | 0.3 | 100 | 39.2 |
|  | *lacI* | n/c | 98.9 | 6.9 | 99.1 | 100 | 98.7 | 98.5 | 99.6 | 99.4 | 99.7 | 0.0 | 60.8 |
|  | *lacO1* | n/c | 1.1 | 49.0 | 0.0 | 0.0 | 0.0 | 0.0 | 0.0 | 0.0 | 0.0 | 0.0 | 0.0 |
| n/c = not counted. Population was contaminated and excluded | | | | | | |  |  |  |  |  |  |  |
